# Overcoming T cell tolerance to tumor self-antigens through catch-bond engineering

**DOI:** 10.1126/science.adx3162

**Published:** 2026-03-19

**Authors:** Xiaojing Chen, Zhiyuan Mao, E. Motunrayo Kolawole, Margherita Persechino, Kevin M. Jude, Masato Ogishi, Kelvin C. Mo, Jami McLaughlin, Donghui Cheng, Xinyu Xiang, Xinbo Yang, Caitlin Gee, Shiqin Liu, Aerin Yang, Matthias Obenaus, Nan Wang, Miyako Noguchi, Tanya Stoyanova, John K. Lee, Zinaida Good, Naomi R. Latorraca, Brian D. Evavold, Owen N. Witte, K. Christopher Garcia

**Affiliations:** 1 Department of Molecular and Cellular Physiology, Stanford University School of Medicine; Stanford, CA 94305, USA; 2 Department of Microbiology, Immunology, and Molecular Genetics, University of California, Los Angeles (UCLA); Los Angeles, CA 90095, USA; 3 Department of Molecular and Medical Pharmacology, UCLA; Los Angeles, CA 90095, USA; 4 Department of Pathology, University of Utah School of Medicine; Salt Lake City, UT, 84132, USA; 5 Department of Biochemistry and Molecular Biophysics, Columbia University Irving Medical Center, New York, NY, USA; 6 Department of Medicine, Division of Immunology and Rheumatology, Stanford University School of Medicine; Stanford, CA 94305, USA; 7 Department of Medicine, Center for Biomedical Informatics Research, Stanford University School of Medicine; Stanford, CA 94305, USA; 8 Howard Hughes Medical Institute, Stanford University School of Medicine; Stanford, CA 94305, USA; 9 Department of Urology, UCLA; Los Angeles, CA 90095, USA; 10 Department of Medicine, Division of Hematology/Oncology, David Geffen School of Medicine at UCLA; Los Angeles, CA 90095, USA; 11 Parker Institute for Cancer Immunotherapy, Stanford University; Stanford, CA 94305, USA; 12 Eli and Edythe Broad Center of Regenerative Medicine and Stem Cell Research, UCLA; Los Angeles, CA 90095, USA; 13 Parker Institute for Cancer Immunotherapy, UCLA; Los Angeles, CA 90095, USA; 14 Jonsson Comprehensive Cancer Center, David Geffen School of Medicine at UCLA; Los Angeles, CA 90095, USA; 15 Molecular Biology Institute, UCLA; Los Angeles, CA 90095, USA; 16 Department of Structural Biology, Stanford University School of Medicine; Stanford, CA 94305, USA

## Abstract

T cells are often weakly responsive to tumor self-antigens because of central tolerance, constraining their ability to eliminate tumors. We exploited mechanical force to engineer a weakly reactive TCR specific for a non-mutated tumor associated antigen (TAA), Prostatic Acid Phosphatase (PAP). We identified a catch-bonding “hotspot” whose mutation enhanced T cell activity by increasing TCR-pMHC bond lifetime, whilst preserving physiological affinities and antigen fine-specificities. T cells expressing these engineered TCRs showed vastly superior expansion in the tumor, effector phenotypes and tumor elimination. Crystal structures and molecular dynamics simulations reveal a single amino acid mutation at the catch bond hotspot primes the TCR for peptide interaction through water reorganization at the TCR-pMHC interface. Catch bond engineering is a viable biophysically-based strategy for transforming tolerized anti-tumor T cells into potent TCR-T killers.

## Introduction

Immunotherapies utilizing T cells as major effectors to treat cancer have proven to be clinically successful for a subset of patients at present. Examples include the chimeric antigen receptor (CAR) T therapy targeting CD19 for treatment of B cell malignancies, check point inhibitors (CPIs) that revive T cells from exhaustion and are broadly applicable for various cancer types and more recently, CARv3-TEAM-E T cells targeting EGFRvIII variant to treat recurrent glioblastoma (1-7). Adoptive T cell receptor (TCR)-T therapy (TCR-T) is an attractive alternative to CAR-T because of the high sensitivity of T cells to low density antigens, and also the potential tumor selectivity of peptide-major histocompatibility complex (pMHC) targets. Afamitresgene autoleucel, a TCR-T therapy targeting MAGE-A4, was recently approved by the Food and Drug Administration (FDA) in August 2024 as the first therapy in this category to treat synovial sarcoma (8-10). Numerous TCR-T cell therapies are currently undergoing clinical evaluation to treat cancer.

A major challenge hindering the application of TCR-T cell therapies in clinical settings is the low potency of patient-derived TCRs against cancer antigens, since a large category of cancer antigens are self-antigens known as tumor associated antigens (TAAs). TAAs are becoming attractive targets for developing new T cell-mediated immunotherapies due to their broader expression, which can cover a larger cohort of patients. Yet, central tolerance places a natural constraint on the ability of T cells to kill non-mutated TAA by deleting highly cytotoxic T cells against such antigens (11, 12). TCRs against TAAs usually require further engineering to improve the *in vivo* efficacy of the T cells (12). While affinity maturation can indeed improve the tumor killing properties of TCR-T, it can change the way the TCR sees the pMHC, which carries the risk of off-target reactivity and clinical toxicity (13-17). An affinity matured TCR-T to the MAGE-A3 antigen led to extreme toxicity by cross reactivity to an epitope of a cardiac antigen (18, 19). The process of affinity maturation changes the free energy landscape for how natural TCRs have been evolved for exquisite antigen specificity through low affinity and broadly distributed free energy landscapes (20, 21). In contrast affinity maturation can focus TCR binding to high affinity hotspots on pMHC that predisposes them to self-reactivity (17, 22).

The interaction between a TCR and its cognate pMHC is typically low affinity, falling within the range of 1-100 μM (17, 23, 24). Yet, T cells are highly sensitive and can detect as low as a single pMHC molecule on the target cell surface and can distinguish antigens that differ by just one amino acid (24-30). The TCR is activated by pMHC through a mechanosensory mechanism known as a “catch bond,” in which the lifetime of a receptor-ligand interaction is prolonged under mechanical force (31-34). TCR activation can be decoupled, to some degree, from binding affinity by means of catch bonds (agonist peptide) versus slip bonds (non-agonist peptides) (35). We previously exploited this triggering mechanism to engineer the MAGE-A3 specific TCR with enhanced *in vitro* activation sensitivity whilst maintaining physiological affinity and showing no discernible off-target reactivity with TITIN, as was seen with the clinically deployed affinity-matured TCR (36). While this study demonstrated that TCR potency could be enhanced by catch bond engineering *in vitro*, it did not show that this strategy could transform tolerized T cells into effective anti-tumor T cells *in vivo*, nor did it provide structural clarity for the mechanism.

Here we exploit the natural mechanosensory mechanism of TCR activation to apply the concept of catch bond engineering of TCR-T to a prostatic acid phosphatase (PAP) specific TCR as a means of overcoming central tolerance (21). High serum PAP levels correlate with a worse prognosis of prostate cancer (37, 38). The expression pattern of PAP is restricted to benign prostate luminal cells and prostate cancer, making it an excellent TCR therapeutic target for prostate cancer patients who have undergone radical prostatectomy—a procedure that removes the entire prostate gland (39). Previously, we identified a TCR (TCR156) from patient PBMC recognizing a physically defined epitope of PAP on HLA-A*02:01 (40). TCR156 expressing T cells exhibited low potencies, suggesting that TCR engineering is required before application of the TCR156 in the ACT. We were able to dramatically enhance the sensitivity of the PAP-specific TCR156 through a newly developed catch bond engineering workflow, to show effective cytotoxicity at low effector to target ratios, superior effector function, and to enable elimination of tumors *in vivo*. The anti-tumor activities of the ‘turbocharged’ TCR correlated better with bond lifetimes than 3D affinity. We resolved, at high resolution, the structural disposition of the catch bond hotspot at the TCR/pMHC interface. Molecular dynamics shows water reorganization primes the engineered TCR to form new peptide interactions, providing a mechanistic rationale, at atomic resolution, for the enhanced in vivo anti-tumor efficacy.

## Results

### TCR turbocharging: A pipeline to engineer catch bond hotspots without prior structural information

TCR156 was isolated from human PBMCs and recognizes an epitope of PAP (PAP_22:_ TLMSAMTNL) restricted on HLA-A*02:01 (40). Despite its ability to secrete small amounts of IFN-γ upon full length PAP expression on an A2^+^ K562 line, it failed to kill the prostate cancer line PC3 that is transduced to express HLA-A*02:01 (HLA-A2) and full-length PAP (PC3-PAP-A2), showing the low potency of the TCR. Despite its specificity in recognizing the processed PAP epitope, TCR156 would not be a suitable candidate for TCR-T therapy of prostate cancer, so we nominated TCR156 for catch bond engineering. We wished to augment the TCR156 functional response to the tumor antigen, whilst retaining physiologically low affinity and fast off-rate, so we turned to catch bond engineering. Since we lacked any structural information, we developed a three-step catch bond engineering workflow that does not require structural information and is generalizable to any TCR: 1- CDR hotspot scanning, 2- Gene shuffling CDR hotspots, and 3- Site saturation mutagenesis.

**Fig. 1:**
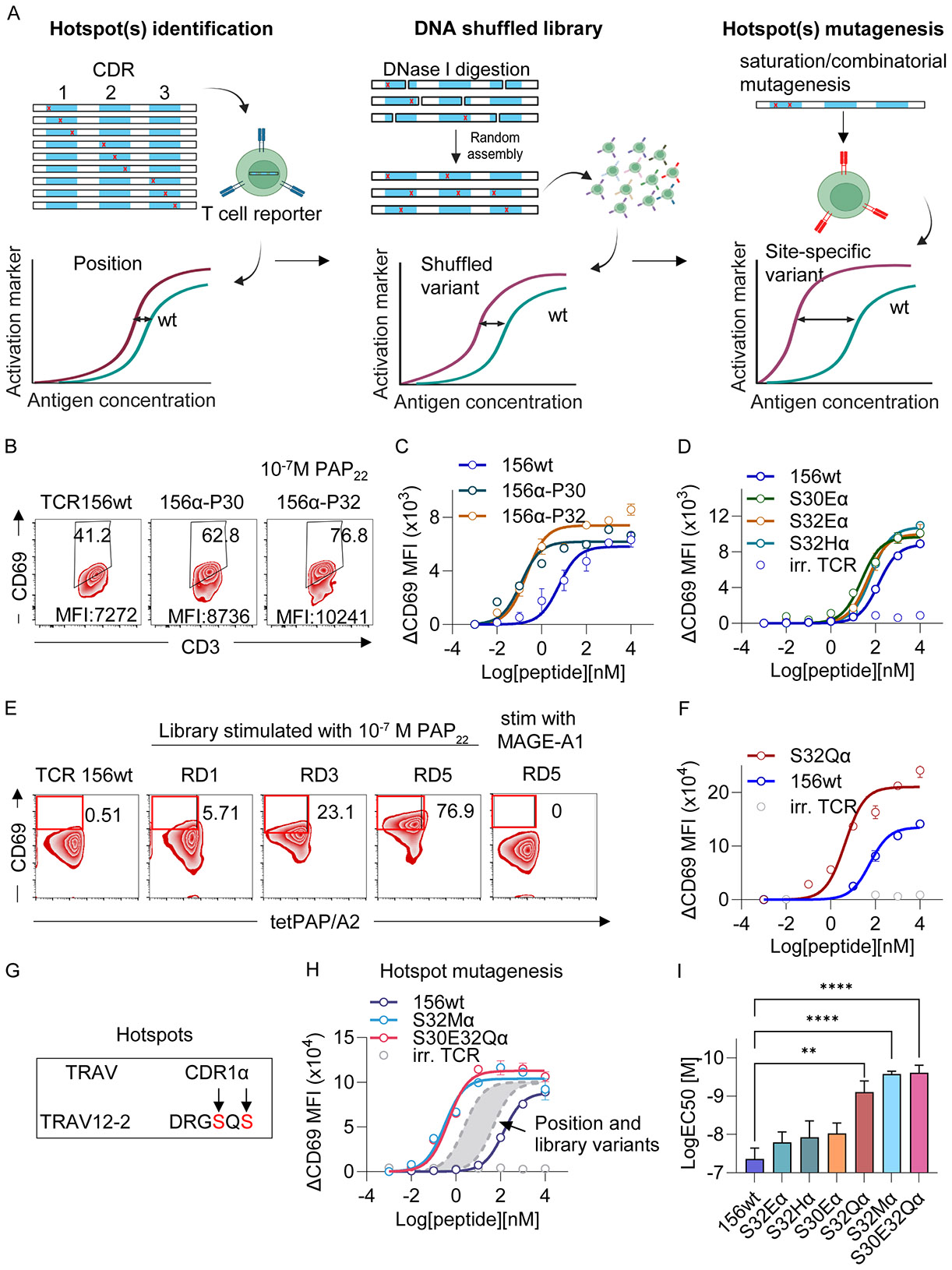
TCR catch bond engineering improves the sensitivity of the PAP_22_/HLA-A2 specific TCR156. (**A**) Schematic illustration of the strategy for PAP TCR engineering. Three major steps were designed, each intended to further augment the TCR156 potency. (**B-D**) Position scanning of TCR156α. (**B**) Example FACs plot of CD69 expression of SKW-3 cells expressing TCR156 bearing mutations at position 30 and 32 compared to TCR156wt upon stimulation with 10^−7^M PAP_22_. (**C**) Titration curves of the TCR156 mutants that showed enhanced CD69 expression upon antigen stimulation. (**D**) Deconvolution of position 30 and 32. The experiments were carried out in duplicate, each experiment was repeated once. (**E-F**) DNA shuffling library selection. (**E**) SKW-3 cells expressing the TCR156α DNA shuffling library were selected for five rounds to enrich population that expressed higher CD69 but no increased binding to PAP_22_/HLA-A2 tetramer compared to the wild-type TCR156 SKW-3 cells. Round 1, 3, and 5 selection are shown. The round 5 selected skw3 T cells did not respond to MAGE-A1/HLA-A2 stimulation (last plot). The selected cells were deep-sequenced and we test the top 12 clones. Peptide titration curve for the best clone S32Qα is shown in (**F**). (**G**) Illustration of the two “catch-bond hotspot” on TCR156α CDR1 domain. (**H**) The hotspot Ser32 was substituted with the additional 16 amino acids. The amino acid substitutes that enhanced the EC50 of the TCR upon peptide titration were then combined with the Ser30Glu mutation to screen for EC50 enhanced clones. The S32Mα and the S30ES32Qα TCR mutants were shown in (H). The grey area indicated the distribution of all the other TCR mutants that exhibited EC50 enhancement during the previous selections. These experiments were repeated once, each experiment included duplicates. (**I**) The EC50 comparison of the EC50 improved clones from different selection steps shown as Mean ± SEM. ONE-way ANOVA followed by Dunnett for multiple comparisons was performed to compare TCR156 variants to 156wt. * P ≤ 0.05; ** P ≤ 0.01; *** P ≤ 0.001; **** P ≤ 0.0001. These experiments were repeated twice, each experiment included duplicates. Abbreviation: irr. TCR: irrelevant TCR. The baseline CD69 MFIs from the titration curves, which are SKW-3 cultured with T2 cells without peptides, were subtracted.

In the first step, we systematically scanned every amino acid on the CDR loops of the TCR156 for potency-enhancing mutations (Fig. 1A). Specifically, we substituted the TCR156 CDRs with polar amino acids Asparagine (N), Histidine (H) or Glutamic acid (E). These amino acids were chosen since their side chains contain functional groups that can potentially form multiple H bonds with their interacting partners, hence increasing the likelihood to form catch bonds with the PAP/HLA-A2 molecule. We selected the TCR156 mutants that did not significantly increase affinities between the TCR and the PAP/HLA-A2 molecule to minimize the chance of direct bond formation between the mutations and the PAP/HLA-A2, and to reduce the risk of generating unexpected cross reactivity to the HLA-A2 molecule.

We constructed positional scanning libraries with N/H/E mutations replacing each residue on the CDRs of either the TCR156α or β chain and introduced the libraries into the SKW-3 reporter line (41). Among all the T cell libraries, SKW-3 cells bearing mutations at position 30 (p30α) and 32 (p32α) on the TCRα chain CDR1 (CDR1α) exhibit escalated maximum CD69 MFI (Fig. 1B) and enhanced sensitivity (Fig. 1C) compared to wild type TCR156 upon stimulation with the PAP_22_ peptide presented on T2 cells (fig. S1A and B for the screening of all positions on both TCRα and β chains). Deconvolution of the two TCRα libraries indicates that the E variant at 30α (S30Eα), and E/H (S32Eα and S32Hα) at 32α were the major contributors to the increased potency of the TCR (Fig. 1D, also see fig. S1C and D for deconvolution of all positions that show enhanced recognition compared to wild type TCR156).

The identified ‘hotspot’ positions from CDR scanning were subjected to additional coarse-grained mutagenesis to further enhance the functional properties of the TCR mutants. The position mutants were used as input for gene shuffling to generate TCR156α or β DNA shuffled libraries. We use codons ‘CAC’ for H, ‘AAT’ for N and ‘GAG’ for E, which by shuffling can generate H (‘CAC’,’CAT’), Q (‘CAG’), N (‘AAC’,’AAT’), K (‘AAG’), D (‘GAC’,’GAT’) and E (‘GAG’) with random recombination of the nucleotides. The theoretical diversities of the libraries were 287,280 for TCRα and 437,184 for TCRβ chain, assuming that there are up to 3 mutations on each construct. We generated TCRα and TCRβ libraries that covered 10x of the theoretical diversity and introduced them into 3x10^7^ SKW-3 cells. We stimulated the library transduced reporter cells with 10^−7^M PAP_22_ peptide presented by T2 cells for 14h, and sorted for cells that upregulate higher CD69 than the wild type. To avoid selection of high affinity clones of the variants, the library cells that stained higher MFI of the PAP_22_/HLA-A2 tetramer than the unmodified wild type TCR156 were gated out. After five rounds of selection, a group of CD69^hi^ and tetPAP_22_/HLA-A2^lo^ SKW-3 cells were enriched for the TCRα library (Fig. 1E). These cells did not cross react to an irrelevant antigen (i.e. MAGE-A1) (Fig. 1E **right panel**). The sorted TCRα library cells were subjected to next generation sequencing (NGS) together with the beginning library (fig. S2A). Interestingly, the dominant mutations are located on 30α and 32α, replicating the results of the CDR positional scanning experiment (fig. S2B). Additional prevalent mutations are R28Q on CDR1, G55N/H on CDR2 and R96H on CDR3 (fig. S2B). We tested the top 12 TCR156 variants from the selected library (fig. S2C). The variant S32Q (S32Qα) exhibited further enhancement of maximum CD69 MFI and EC50 in comparison of the wild type TCR156 and the TCR S30Eα, S32Eα/Hα variants (Fig. 1F, fig. S2D).

Based on the observation that several potency enhancing mutations (Glu, His, Gln) of TCR156 are located on the CDR1α chain at position 32 (Fig. 1G), we sought to further improve the TCR function by saturation mutagenesis with all amino acids at this position (except C). Nineteen TCR156 variants carrying different amino acids at this position were introduced into the SKW-3 reporter line individually to assess their potencies. Among the variants, the methionine mutation (S32Mα) demonstrated enhanced functionalities based on the CD69 expression level after PAP antigen stimulation (Fig. 1H, fig. S3A). As the final step, we combined the E mutation from position 30 with E, H, M, Q mutations on position 32 and tested their functionalities in the SKW-3 reporter cells. All the combinatorial mutations exhibit improved TCR potencies, especially for the combination of E at position 30 and Q at position 32 (S30E32Qα) (Fig. 1H, fig. S3B). Although the mutant S30E32Mα showed the highest TCR potency enhancement, it shows some cross-reactivity to an HLA-A2^+^ cell line without the presence of PAP antigen and hence was removed from further analysis (fig. S3C).

The “turbocharged” TCR156 variants from different schemes showed a stepwise improvement in functional EC50’s spanning 2 logs (Fig. 1I). This sequential increment of TCR potency was confirmed in primary human T cells transduced with the TCR156 variants, while comparing their responses upon stimulation with the PAP_22_ peptide, which includes the expression of CD107a, the serine protease Granzyme B, cytokines IFNγ and TNFα (fig. S4).

### Functional potency of TCR mutants correlates with the TCR-PAP/A2 bond lifetime

**Fig. 2:**
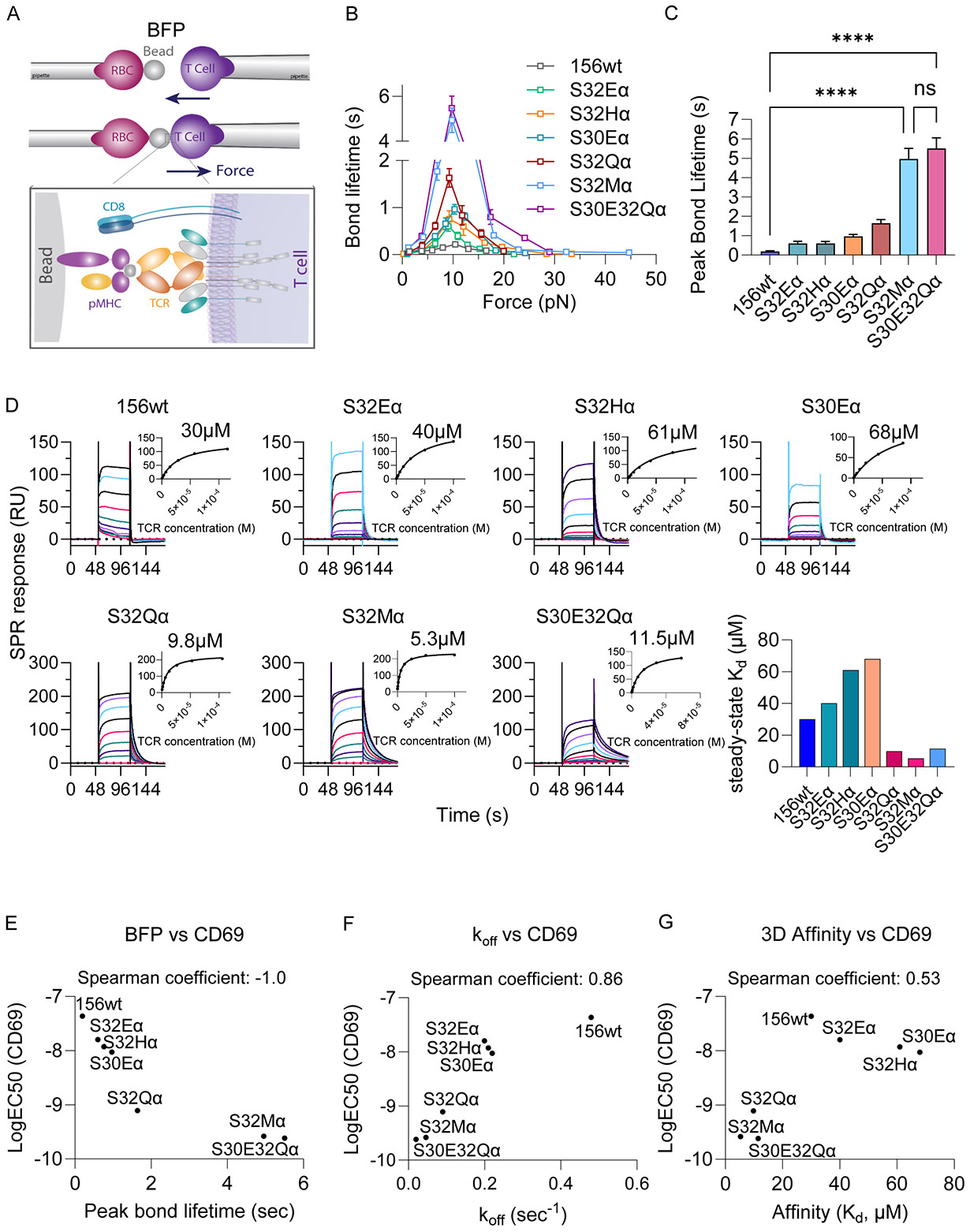
Catch bond engineered TCRs exhibit prolonged bond lifetimes that correlate with enhanced functionality. (**A**) Illustration of the BFP experiment setup. (**B**) Selected TCR variants were subjected to BFP measurement. The bond lifetime between the TCR and PAP/HLA-A*02:01 were measured under zero to 45 pN of force. (**C**) Comparisons of peak bond lifetime of the TCR variants measured in BFP. Data indicate mean ± SEM. ONE-way ANOVA followed by Dunnett for multiple comparisons was performed to compare peak bond lifetime of different groups. * P ≤ 0.05; ** P ≤ 0.01; *** P ≤ 0.001; **** P ≤ 0.0001 (**D**) SPR measurement of the TCR variants with the PAP/HLA-A*02:01. The K_d_ values were calculated and shown for each TCR. (**E-G**) Correlation of the CD69 EC50 versus peak bond lifetimes of TCR56wt and TCR variants measured by BFP (**E**), koff by kinetic SPR at low coupling density **(F)** or **(G)** Kd measured by steady-state SPR, Spearman coefficient is calculated and shown (data shown in fig. S6).

The function-based selection isolated more sensitive TCR mutants to the PAP antigen, so we sought to interrogate their biophysical properties by two different methods: 1- The two-dimensional (2D) Biomembrane Force Probe (BFP) method between T cells and pMHC coated beads under applied force, and 2- Three-dimensional (3D) affinity (K_d_) using recombinant TCR extracellular domains by surface plasmon resonance (SPR) under zero applied force. BFP measurements between TCR156 variants and the PAP/HLA-A2 were carried out spanning piconewton (pN) range of pulling forces (42) (Fig. 2A). Prior to the BFP experiments, we normalized the surface expression of CD8 on the TCR156 expressing SKW-3 cells across the TCR156 variants (fig. S5). All of the TCRs exhibited stereotypical catch bond behavior, where bond lifetime increased under tensile force until a peak threshold, at approximately 10pN, where it exponentially decreased. Specifically, wild-type TCR156 exhibited a weak peak bond lifetime of around 0.2 sec under 10 pN of force, which is consistent with its weak activation (Fig. 2B). S32Eα, S32Hα, S30Eα, S32Qα, S32Mα and S30E32Qα showed significant increases in bond lifetime compared to the wild-type TCR156. S32Mα and S30E32Qα showed the most significantly enhanced catch bonds, which peaked at 10 pN with a bond lifetime of around 5 sec (Fig. 2C).

The 3D affinities by SPR range from K_d_ 5.3μM (S32Mα) to 68μM (S30Eα), with the wild-type TCR156 showing an affinity of 30μM (Fig. 2D, fig. S6A). Overall, the potency of activation of TCR156-expressing SKW-3 T cells shows a stronger correlation with the 2D bond lifetime than 3D K_d_ (Fig. 2E and G), whilst maintaining physiological TCR-pMHC affinity ranges (K_d_ ~5-50uM) that limit the potential for off-target cross-reactivity. Bond lifetime measured under force is proportional to the off-rate (k_off_) of bimolecular interactions measured at zero force by SPR. Spearman analysis shows that the rank order of TCR potency correlated well with k_off_ measured by SPR, but bond lifetime under force showed an improved correlation (Fig. 2F). Since T cells see pMHC under approximately 10pN shear force, bond lifetime measured under applied force on T cells reflects our activation-based selection strategy that was designed to preserve fast off-rate while augmenting bond lifetime.

### TCR156 catch bond mutants exhibit significantly improved functions in primary human T cells

**Fig. 3:**
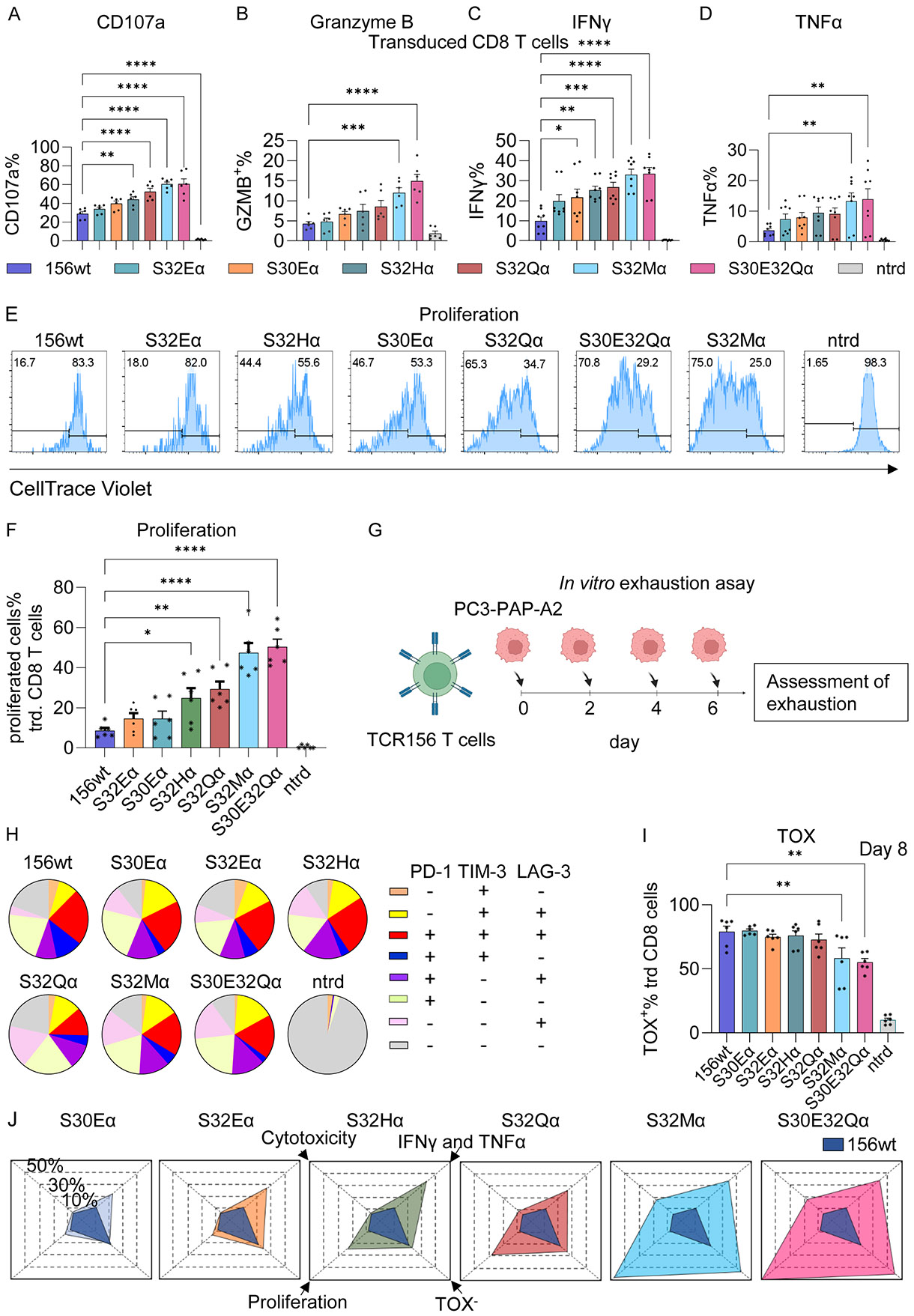
T cell profiling of the TCR156 variants. (**A**) Membrane expression of CD107a, the cytotoxic degranulation marker, on primary human T cells after the T cells were cultured with PC3-PAP-A2 for 4 hours at an E:T ratio of 4:1. The experiments were performed in duplicates and repeated at least once with human PBMCs from 3 donors. Data indicate mean ± SEM. (**B-D**) Primary human T cells expressing TCR156 variants were co-cultured with PC3-PAP-A2 for 16 hours at an E:T ratio of 4:1. Intracelluar staining was performed to evaluate the production of Granzyme B (B), IFNγ (**C**) and TNFα (**D**). Experiments were performed in duplicates and repeated at least once with human PBMCs from 3-4 donors. Data indicate mean ± SEM. (**E-F**) T cell proliferation upon stimulation with PC3-PAP-A2 for 72 hours at an E:T ratio of 4:1. The T cells were labeled with CellTrace dye and the proliferated cells and the number of proliferation cycles were tracked by flow cytometry. Representative plots are shown in (**E**). The percentage of original T cells that underwent proliferated were compared in (**F**). Experiments were performed in duplicates with human PBMCs from 3 donors. Data indicate mean ± SEM in (**F**). (**G-I**) *in vitro* exhaustion assay with repetitive PAP antigen stimulation. 2x10^4^ PC3-PAP-A2 cells were added every 2 days to a starting culture of 4x10^4^ primary T cells with TCR156 variants as illustrated in (**G**). The exhaustion states were determined on day 7-8. (**H**) Percentage of different IRs were depicted in Pie charts. (**I**) The expression of the transcription factor TOX in the T cells 12 days after repetitive stimulation. The exhaustion assay was performed with 3 human donors, each in duplicates. ONE-way ANOVA followed by Dunnett for multiple comparisons was performed to compare the TCR156 variants to 156wt for plots A-D, F and I. * 0.01< P ≤ 0.05; ** 0.001< P ≤ 0.01; *** 0.001< P ≤ 0.001; **** P ≤ 0.0001 (**J**) Radar plot showed the overall improvement of cytotoxicity (represented by Granzyme B), IFNγ and/or TNFα, proliferation potency and resistance to exhaustion (TOX^−^ T cells) of the TCR156 mutants compared to wild-type TCR. Abbreviation: ntrd: non-transduced

We evaluated the immune response profiles of the TCR156 mutants with a prostate cancer line, PC3-PAP-A2, in which the PAP_22_ epitope is naturally processed and presented by HLA-A2 on the cell surface. At an effector-to-target (E:T) ratio of 1:4 (**see** fig. S7 **for additional E:T ratios**), we observed a stepwise increase in the percentages of cells expressing the degranulation marker CD107a on the cell surface with the same hierarchy established in the previous CD69 upregulation experiment (Fig. 3A). Notably, only the two most potent TCR156 mutants, S32Mα and S30E32Qα, exhibited a significantly higher frequency of cells that produced Granzyme B, one of the key molecules which mediates apoptosis in target cells (43) (Fig. 3B).

IFNγ and TNFα are the two major pro-inflammatory cytokines that are secreted by cytotoxic T cells in response to antigen stimulation. Not only do they mediate direct T cell killing of the target tumor cells, they also play critical roles in modulating the tumor microenvironment (44-47). Higher percentages of the TCR156 mutant expressing T cells secreted IFNγ and TNFα compared to TCR156 wild-type T cells, with the S32Mα and S30E32Qα T cells demonstrating statistically significant enhancements in cytokine productions compared to TCR156 wild type (Fig. 3C and D).

The capacities of T cells to undergo clonal expansion when encountering tumor cells is one of the decisive factors in tumor clearance during ACT. We monitored T cell proliferation during co-culture with the PC3-PAP-A2 cells for 72 hours. The TCR mutants resulted in a greater percentage of proliferating T cells. The responding cells underwent more rounds of divisions, as indicated by the peaks associated with different CellTrace Mean Fluorescent Intensities (MFIs) (Fig. 3E, fig. S7E). We calculated the percentage of initial T cell population that divided upon encountering the antigen. As expected, S32Mα and S30E32Qα T cells demonstrated significantly greater proliferation compared to the wild-type TCR156, while the rest of the TCR mutants showed improved proliferation capacities, albeit not to a statistically significant extent (Fig. 3F).

In order to evaluate whether the improved proliferation would alter the exhaustion signatures of the TCR156 mutants expressing T cells, we repetitively stimulated the TCR156 variant expressing T cells with PC3-PAP-A2 cells for 7 days, adding a new batch of the target cells every two days (Fig. 3G). More than 50% of the T cells exhibited an exhausted phenotype, characterized by the upregulation of inhibitory receptors such as PD-1, LAG-3 and TIM-3, and reduced capacity to produce cytokines IFNγ and TNFα, as well as the upregulation of transcription factor TOX (fig. S8). Interestingly, the more functionally responsive TCR156 mutants did not co-express escalated levels of multiple inhibitory receptors compared to the wild-type TCR. For example, we did not observe higher percentages of PD-1^+^TIM-3^+^ (colored blue in the pie diagrams) double positive or PD-1^+^TIM-3^+^LAG-3^+^ (colored aqua in the pie diagrams) triple positive T cells in the catch bond engineered TCR T cell populations compared to wild-type T cells (Fig. 3H). Nearly 90% of the wild-type TCR156 T cells upregulated the transcription factor (TF) TOX, which is a marker associated with T cell dysfunction and exhaustion (48-50). In contrast, the TCR156 catch bond exhibited an attenuated phenotype, with progressively fewer cells upregulating TOX, particularly in the groups of TCR S32Mα and S30E32Qα compared to the wild-type T cells (Fig. 3I). Our analysis reveals that the TCR catch variants have improved immune profiles concordant with increased bond lifetime and correspondingly reduced k_off_ compared to TCR156wt both in cytotoxicity functions, the proliferation potency and resistance to exhaustion (Fig. 3J).

### TCR-pMHC bond lifetime predicts TCR-T anti-tumor activity

**Fig. 4:**
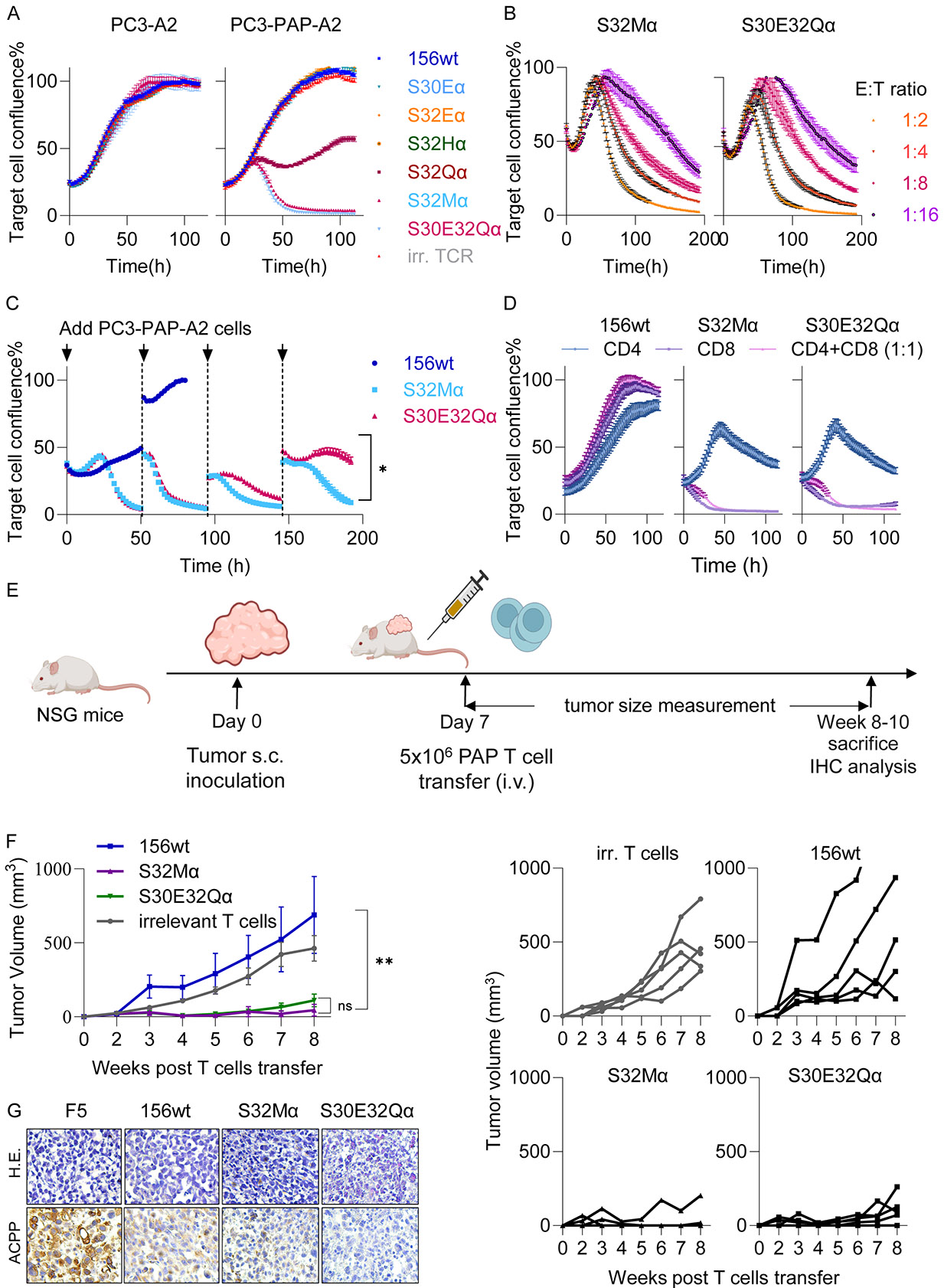
Anti-tumor potencies of the TCR156 variants. (**A-D**) *In vitro* killing of the TCR156 variants expressing human PBMCs measured in Incucyte assay under different conditions. (**A**) E:T ratio of 1:1; (**B**) Different E:T ratios as indicated were tested for TCR156 variants S32Mα and S30E32Qα; (**C**) TCR156 variants S32Mα and S30E32Qα were stimulated by PC3-PAP-A2 repetitively every 2 days. (D) Transduced CD4 and CD8 T cells expressing wt, S32Mα, S30E32Qα TCRs were cultured with PC3-PAP at E:T ratio of 1:1. These experiments were repeated using PBMCs from at least three healthy individuals; each experiment included triplicates. (**E**) Timeline of adoptive T cell transfer in NSG mice. (**F**) Tumor size measurement of mice treated with different TCR expressing human PBMCs either as average (left panel) or individual mice (right panels). (**G**) Immunohistochemistry staining of the excised tumor samples after adoptive T cell transfer. The tumor experiment was repeated in three independent experiments using PBMCs from three healthy individuals, each experiment included 5 animals per group.

To determine the relative abilities of wt TCR156 versus catch bond mutants in target cell killing, we cultivated the T cells with PAP expressing PC3-PAP-A2 cells at 1:1 E:T ratio in an Incucyte assay. These analyses dynamically tracked the fluorescence area over 5-10 days to estimate the killing activity of PBMCs. No significant off-target effects were observed on PC3-A2 cells lacking PAP expression (Fig. 4A **left**). Three modified TCR candidates, S32Qα, S32Mα, and S30E32Qα, demonstrated enhanced cytotoxicity against PC3-A2-PAP cells (Fig. 4A **right**). Notably, S32Mα and S30E32Qα TCRs showed sustained and more pronounced killing effects compared to S32Qα (Fig. 4A **right**). To assess sensitivity and potency under conditions where cancer cells were outnumbered, we set up co-culture experiments with various E:T ratios (1:2, 1:4, 1:8, 1:16). S32Mα and S30E32Qα exhibited significant cytotoxic effects even at the lowest E:T ratio (1:16), indicating highly potent reactivity (Fig. 4B). Furthermore, repetitive cancer cell challenge experiments were conducted by introducing fresh PC3-A2-PAP cells every 48 hours. Both S32Mα and S30E32Qα TCR-modified PBMCs maintained cytotoxicity across four tumor challenge cycles, suggesting prolonged *in vitro* killing effects (Fig. 4C). Notably, S32Mα demonstrated more efficient killing after the third coculture cycle compared to S30E32Qα (Fig. 4C). The cytotoxicity is mainly driven by the engineered CD8 T cells, as limited tumor killing was observed when only transduced CD4 T cells were present (Fig. 4D).

To better assess the systematic effects and long-term tumor surveillance of catch bond-engineered TCRs *in vivo*, we further evaluated them using tumor models engrafted in immunodeficient NSG mice. PC3-PAP-A2 cells were allowed to grow in NSG mice for 7 days before the T cells were transferred intratumorally (fig. S9A). Wild-type TCR156 failed to control the outgrowth of the PC3-PAP-A2 tumor. Despite the similar 3D affinities between TCR S32Qα, S32Mα and S30E32Qα to PAP/HLA-A2, TCR S32Qα was less competent in controlling tumor growth compared to the other two TCRs, and 2 out of 6 mice developed tumors. In contrast, S32Mα and S30E32Qα delayed or completely inhibited tumor growth during a time course of 8 weeks (fig. S9B).

The augmented potencies of S32Mα and S30E32Qα T cells in tumor control was further demonstrated in another *in vivo* adoptive T cell treatment setting, in which the T cells were transferred intravenously into the PC3-PAP-A2 tumor bearing NSG mice (Fig. 4E). S32Mα and S30E32Qα T cells prevented tumor outgrowth over the course of measurement (8 weeks), which is significantly different from the wild-type TCR156 T cell treated groups, in which the T cells failed to control the tumor growth (Fig. 4F). No statistically significant difference was found between the groups introduced with either wild-type TCR156 or an irrelevant TCR (DMF5, or F5) previously used for clinical studies targeting MART-1 antigen in melanoma (51) (Fig. 4F). The immunohistochemistry (IHC) staining of the tumor tissue with PAP antigen at the end point of the experiment suggested that the remaining tissue expressed high levels of PAP (also known as ACPP) in the negative control (F5) and wild-type TCR156 groups. In contrast, tumor cells showed reduced or absent expression of PAP in the S32Mα and S30E32Qα treatment groups (Fig. 4G).

### Enhanced proliferation and effector functions in intratumoral TCR-T cells

**Fig. 5:**
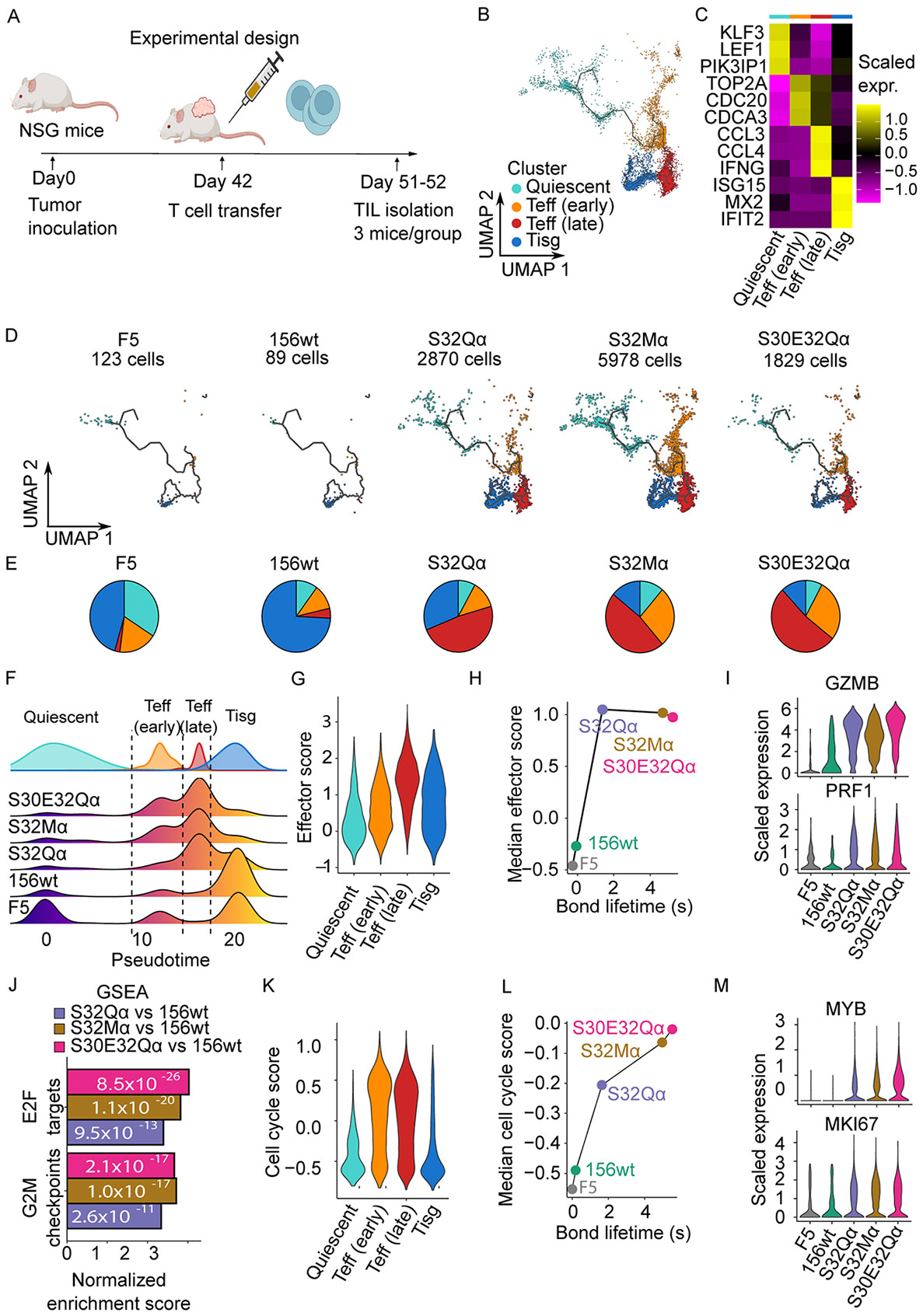
Single-cell RNA-sequencing analysis of the TILs from the PC3-PAP-A2 xenograft in NSG mice. (**A**) Experimental setup for isolation of TILs from different groups of T cells transduced with non-engineered (i.e., wild-type; TCR156) or three variants of catch bond engineered TCRs. An irrelevant TCR (F5) was also used as a negative control. For each experimental group, TILs collected from three individual animals were pooled (n=3) and sequenced. (**B**) Uniform Manifold Approximation and Projection (UMAP) representation of TILs from all groups combined. The TILs sequenced in this study were mapped onto a reference pan-caner T-cell atlas (52) to cluster the cells according to their differentiation states. (**C**) Heatmap of the representative genes for each cluster. (**D**) UMAP of individual groups. (**E**) Pie chart indicating the proportion of each cluster in individual groups. (**F**) The distribution of different clusters on the pseudotime axis for the different TIL groups. (**G**) The effector scores for each clusters (see methods for effector score calculation). (**H**) Correlation of the BFP peak bond lifetime and the median effector scores of different TCR156 variants. (**I**) Scaled expression levels of representative genes for T-cell effector function. (**J**) Geneset enrichment analysis indicated the pathway differences between the TCR catch-bond engineered TILs and the wild-type TILs were E2F targets and G2M checkpoints. (**K**) The Cell-cycle scores for each cluster. (**L**) Correlation of the BFP peak bond lifetime and the median Cell-cycle scores of different TCR156 variants. (**M**) Scaled expression levels of representative genes associated with proliferation.

To gain deeper insight into the impact of TCR catch bond engineering on T cell functions during ACT, we adoptively transferred pre-activated human T cells transduced with TCR156 or its variants at a late time point (42 days post tumor inoculation), in contrast to previous experiments (7 days post inoculation). The tumor infiltrating lymphocytes (TILs) were isolated 9-10 days after the transfer and sorted for the surface expression of the transduction marker NGFR and human CD3, while anti-mouse CD45 was used to exclude mouse immune cells (muCD45^−^huCD3^+^NGFR^+^) (Fig. 5A, fig. S10A). TILs collected from three individual animals within the same experimental group were pooled, processed, and sequenced. We analyzed the transcriptomic profiles of sorted TILs from different groups through single-cell RNA-sequencing (scRNA-seq). After quality control filtering, a total of 10,616 cells were retained for downstream analysis. Much higher numbers of TILs were recovered in the three engineered TCR groups compared to wild-type or F5 negative control groups (Fig. 5D).

We began by comparing the differences in gene expressions between the three catch-bond engineered TILs and the wild-type TCR156 TILs (fig. S10B). IFN response genes (e.g. *IFIT1, IFIT3, ISG15*) or quiescent genes (e.g. *LEF1, CCR7, IL7R*) were upregulated in the wild-type TILs, whereas genes that are critical for effector function (e.g. *GZMB, GNLY*) and proliferation (e.g. *MYB*) were upregulated in the three catch-bond engineered groups (52-55).

To further investigate the differentiation states of the TILs from different groups, we projected our scRNA-seq data onto an existing pan-cancer human TIL atlas (56), followed by an unsupervised clustering analysis (Fig. 5B-F, fig. S10C) (Methods). Four distinct phases of TILs were identified: quiescent, early T effector (T_eff_ (early)), late T effector (T_eff_ (late)), and T cells with high interferon stimulated gene (ISG) expression (T_isg_), based on the gene signatures (Fig. 5B and C, fig. S10C). Most of the wild-type TILs were in the T_isg_ state (Fig. 5D and E). To note, sustained ISG expression is associated with terminal differentiation and dysfunctional in human CD8^+^ T cells (57, 58). In contrast, around half of the TILs in the three catch-bond engineered groups were late effector T cells (expressing effector molecules such as *IFNG* or *CCL3/4*, Fig. 5C-E). An increased proportion of the early effector T cells, which are characterized by cell cycle markers such as *TOP2A*, appeared in the S32Mα and S30E32Qα groups (Fig. 5C-E). Pseudotime analysis revealed a differentiation trajectory of TILs starting from quiescent toward terminal differentiation (T_isg_) states, in which catch-bond engineered TCRs, particularly S32Mα and S30E32Qα, apparently prevented T cells from progressing toward terminal differentiation unlike wild-type TCR (Fig. 5F).

Since effector molecules or markers for proliferation showed expression differences (Fig. 5C, fig. S10C), we performed analysis on the effector function and proliferation capacity of these TILs. The effector score was calculated based on the expression levels of six effector molecules *GZMA*, GZM*B*, GZM*H, GNLY, PRF1,* and *IFNG* on a single-cell basis (Methods). T_eff_ (late) showed the highest Effector score, indicating that T_eff_ (late) cells were the main source of T cells exerting the anti-tumor functions (Fig. 5G). The three TCR catch bond-engineered groups showed enhanced effector functions compared to the wild-type TILs. The degrees of enhancement were comparable among the three groups (Fig. 5H and I), implying that the effector function alone does not fully explain the improved anti-tumor effect we observed, particularly for the S32Mα and S30E32Qα groups, *in vivo*. Comparisons of the differences of upregulated pathways using Gene Set Enrichment Analysis (GSEA) revealed the upregulation of genes involved in cell cycle, i.e., G2M checkpoint and E2F targets, in the TCR catch bond-engineered TILs compared to TILs with the wild-type TCR (Fig. 5J), in which the S32Mα and S30E32Qα groups showed even more evident enrichment than the S32Qα group. We then calculated Cell Cycle score based on genes characteristic for the S and G2M phases (Methods). Both T_eff_ (early) and T_eff_ (late) cells exhibited equally high Cell Cycle scores (Fig. 5K), indicating their proliferative potential. Interestingly, the median Cell Cycle score value from each group of the TILs correlates well with the bond lifetime of the TCRs measured by BFP (Fig. 5L and M).

Overall, elongation of bond lifetime of antigen-specific TCR through catch bond engineering rescues intratumoral T cells from rapidly progressing toward dysfunctional states and endows them with enhanced proliferative and effector functionality.

### Structural context of catch bond hotspot mutations

**Fig. 6:**
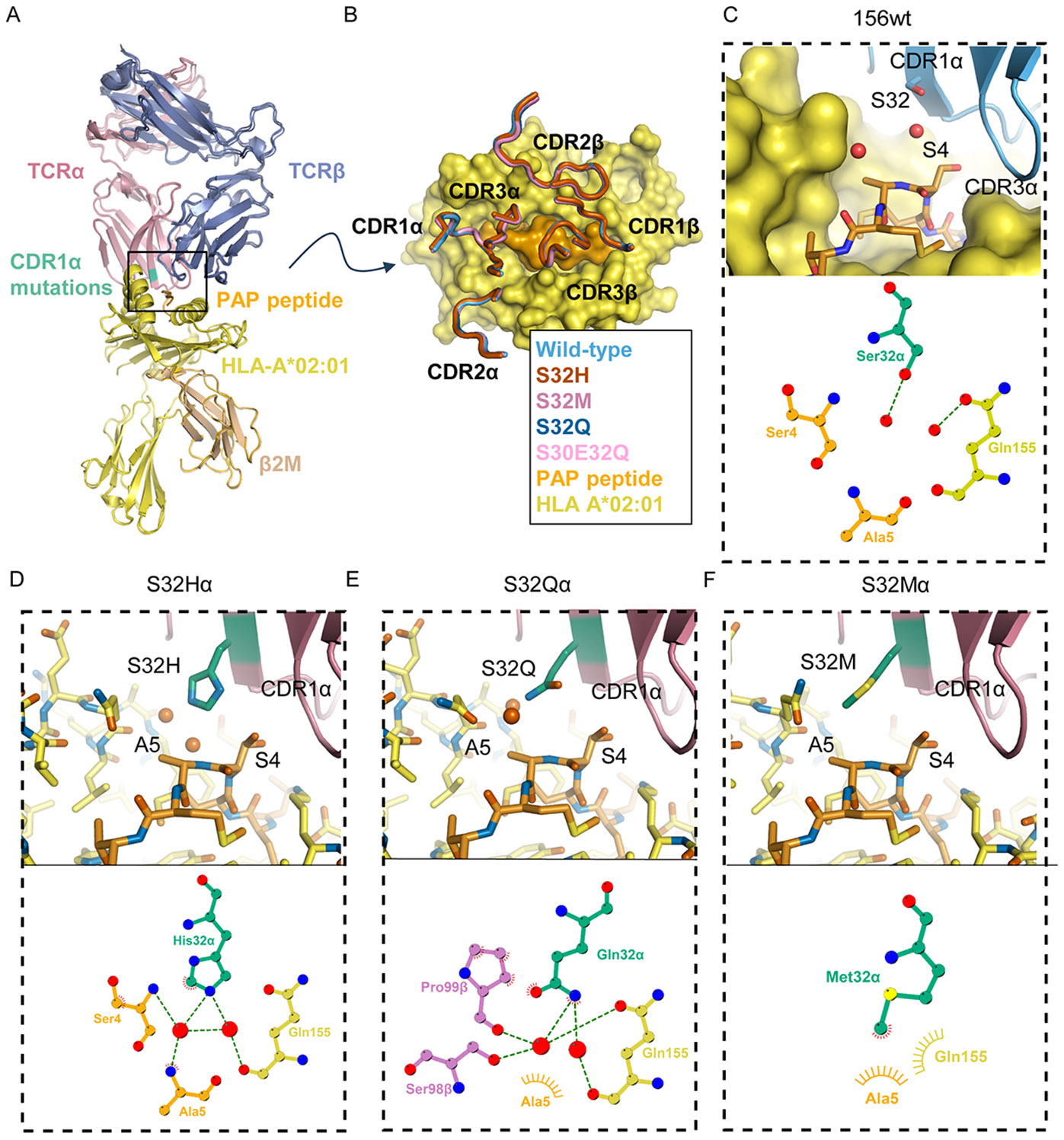
Structural environment of a catch bond hotspot at the TCR-pMHC interface. (**A**) Crystal structures of wild-type TCR156 and mutants superimposed on HLA-A2 (**B**) CDR footprint of TCR156 variants aligned on PAP-A2. Wild-type (sky blue), S32Hα (red), S32Mα (magenta), S32Qα (blue), S30E32Qα (pink) The Cα r.m.s.d. for the TCRs range between 0.298 – 0.369 Å (**C-F**) (top) Details of boxed region in A showing closest approach of Ser32α mutants to PAP_22_ peptide. TCR β chain is omitted for clarity. Bound water molecules resolved in the electron density maps are shown as red spheres. Buried surface area between residue 32 and the peptide/HLA-A2 is: wt: 24 Å^2^; S32H: 160 Å^2^; S32M: 180 Å^2^; S32Q: 153 Å^2^; (bottom) Contact plots of variant residues at position 32 of the alpha chain with coloring as in panel A. Hydrogen bonds are shown as dashed green lines, water molecules as large spheres, and van der Waals contacts are indicated by rays.

Our functional data implicate a principal hotspot site in CDR1α for catch bond acquisition by the 156 TCR, raising the question of the structural role of this position in engaging the PAP/HLA-A2 complex. We determined the crystal structures of the wild-type TCR156, as well as another five TCR mutants, S30Eα, S32Hα, S32Qα, S32Mα and S30E32Qα, in complexes with the PAP/HLA-A2 at resolutions of 1.94-2.19 Å, enabling a very detailed analysis of the structural environment of the interface (table S1). All of the structures superimposed very closely, to within atomic resolution. The mutations on the CDR1α chain did not introduce noticeable changes to the overall TCR-PAP/HLA-A2 tertiary structure, nor did they change the TCR docking angle to the PAP/HLA-A2 complex (Fig. 6A). The contacts between all of the TCR variant CDR residues and PAP/HLA-A2 are virtually identical (Fig. 6B). The CDR1α is located close to the N terminus of the PAP_22_ peptide, but the catch bond hotspot position Ser32 from the wild-type TCR does not have direct contact with the PAP/HLA-A2 complex, and is surrounded by two ordered water molecules, one of which is directly coordinated to S32 (Fig. 6C). In comparison, the side chain of the activating mutant H32 interacts indirectly with the main chain of Gln155 on the HLA-A2 and the side chains of Ser4 and Ala5 on the PAP peptide via water-mediated hydrogen bonds (Fig. 6D). Similar to His32, the Gln32 of an activating mutant formed indirect interactions with Q155 on the HLA-A2, as well as two additional amino acids, Ser98 and Pro99, on the TCR CDR3β chain (Fig. 6E). In contrast, the activating mutant Met32 side chain interacts tenuously with the Ser4 and Ala5 on PAP peptide via van der Waals forces (Fig. 6F). Interestingly, the Glu30 mutation does not locate at the interface between the TCR and the PAP/HLA-A2. Instead, it interacts with the side chain of the neighboring Arg95 on the CDR3α chain via salt bridges (fig. S11A). The mutations at position 32 of TCRα chain increased the buried surface area between the residue 32 and the PAP/HLA-A2 from 24 Å^2^ to up to 180 Å^2^, but this increase did not correlate with the prolonging of bond lifetimes measured by BFP (Fig. 6D-F **upper panels,** fig. S11B). In summary, we do not see evidence in the complex “ground state” for new strong interactions between the mutants at position 32α of the TCR that can clearly explain the acquisition of catch bonds that potentiate the TCR/pMHC interaction to enhance signaling. This is consistent with the notion of catch bonds formed transiently during disengagement. These findings show that catch bond engineering does not simply optimize bond strengths between the TCR and pMHC that lead to higher affinity.

### Probing catch bond mechanism with molecular dynamics simulations

**Fig. 7:**
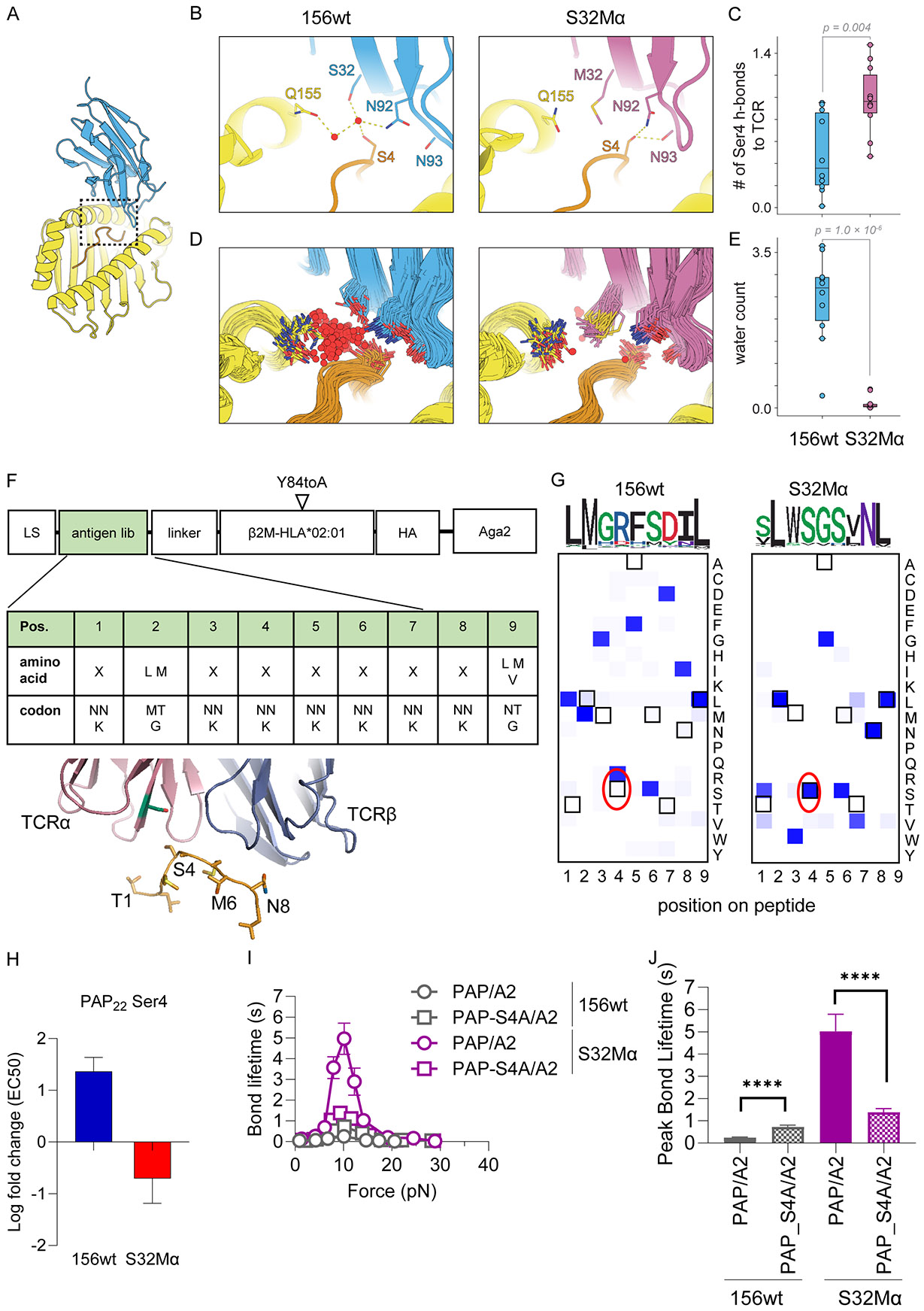
The catch bond hotspot mutation primes the TCR for peptide interaction. (**A-E**) TCR–peptide interface dynamics in MD simulation. (**A**) Inset showing the Vα hotspot region of interest with the TCRβ subunit hidden. (**B**) Representative simulation snapshots show water-mediated hydrogen-bonding networks that form in WT simulations and a network of protein–peptide hydrogen bonds that form in S32M simulations. Note that these protein–peptide hydrogen bonds can also form in WT simulations. (**C**) Box-and-whisker plots show the average number of hydrogen bonds formed between Ser4 and either Asn92 or Asn93 on TCRα at any given frame, averaged across each independent simulation. Ten simulations analyzed for each condition. *P < 0.01* (**D**) Overlay of simulation frames down sampled every 200 ns across ten independent simulations of either complex. Displayed waters are those in a bounding sphere of radius 4.5 Å, centered on the crystallographic Met32 sulfur atom after global alignment of all simulation frames. (**E**) Box-and-whisker plots of the average number of waters within the sphere across ten simulations of either complex. *P < 1 × 10*^*−6*^ (**F-G**) A2-peptide yeast library selection of TCR156wt and S32Mα. (**F**) Yeast library design of HLA-A*02:01 presenting randomized 9mer peptides with restricted amino acids at the anchor position 2 and 9. (**G**) Heat map of round 3 (TCR156wt) or 4 (S32Mα) selected peptides. Darker blue indicates the preferred amino acid at the position. Boxed amino acids represents the PAP_22_ epitope TLMSAMTNL. Serine at position 4 has been circled in red. (**H**) Differences in EC50 for 156wt and S32Mα TCRs when responding to wild-type PAP22 versus PAP22-Ala4. PAP-S4A led to enhanced EC50 for TCR156wt, but reduced EC50 for S32Mα. The changes are shown in logarithmic scale. (**I-J**) BFP measurements of 156wt and S32Mα with the PAP_22_-S4A variant. (**I**) The bond lifetime between the TCRs and alanine variants on PAP/A2 were measured under zero to around 30 pN of force. (**J**) Comparisons of peak bond lifetime of the PAP/A2 variants measured in BFP. Data indicate mean ± SEM. Student T test was performed to compare peak bond lifetime between different groups. **** P ≤ 0.0001

We hypothesized that activating mutations could ‘prime’ certain interactions in the ground state to facilitate catch bond formation under tension. We initiated all-atom molecular dynamics simulations of the TCR156wt and TCR S32Mα/pMHC complexes to visualize the effects of the S32M mutation on peptide–TCR interactions, (see Methods). We carried out ten independent, 1-μs simulations of each structure, for a total of 20 μs of simulation. We visualized residues in the vicinity of Ser32 and found that Ser32 participates in a highly dynamic, water-mediated hydrogen bonding network with Asn92 (TCR-alpha), the backbone of Asn93 (TCRα), Ser4 (peptide), and Gln155 (HLA-A2). Ser4 dynamically forms contacts with Asn92 and Asn93, and we focused our subsequent analysis on these interactions (Fig. 7A-E).

In simulations of S32Mα, the methionine side chain displaces waters from a well-hydrated pocket, separating Gln155 from the Asn92–Ser4–Asn93 network (Fig. 7B-E). We speculated that Met32 may ‘isolate’ this network, increasing the frequency of these TCR–peptide interactions. We determined the interaction strength of the local Asn92–Ser4–Asn93 network by quantifying the number of hydrogen bonds formed between the Ser4 hydroxyl and either TCR residue at each simulation frame (Fig. 7C and fig. S12). The median interaction strength in S32Mα simulations is more than twice that of 156wt, such that, on average, one hydrogen bond to Ser4 is formed throughout the S32Mα simulations. These data suggest that interactions with the peptide may be primed to form in a dynamic process in the activating mutant, even though these same interactions can also form in the WT TCR–MHC complex.

### Catch bond engineered TCR shows enhanced PAP specificity and no off-target cross-reactivities

Cross-reactivity is a major concern in TCR engineering (18, 19). Thus, we carried out a cross-reactivity assessment using a yeast pMHC display library technology that previously successfully identified off-target peptides for an affinity-matured TCR (59). A yeast library expressing 8x10^8^ nonameric peptides presented by HLA-A*02:01 was generated. The amino acids on the peptides were randomized using the codon NNK, with restricted diversity at anchor position 2 and 9 (Fig. 7F). After three to four rounds of selection using TCR tetramers (fig. S13A), heat maps were generated from the deep sequencing data, which did not reveal an increase in cross-reactivity by the engineered TCRs relative to wt. Interestingly, S32Mα appeared to be more selective to PAP_22_, with Ser at position 4 and Asn at position 8 as the primary contact residues (Fig. 7G), indicating that the catch bond engineering enhanced selectivity. While none of the top peptides from the yeast library were represented in the human proteome, we nevertheless predicted 40 peptides from the human proteome that shared similar motifs to random peptides selected by wt TCR or S32Mα, 20 for each. Both the wt and S32Mα showed weak reactivity to a peptide sequence “SLLSSSLNV” from NEDD4 binding protein (N4BP2) (fig. S13B). N4BP2 expresses at high levels in cell lines such as HEK-T or K562 (fig. S13C). Since neither the wild-type nor S32Mα expressing SKW-3 cells could recognize NEDD4 without peptide pulsing, SLLSSSLNV does not appear to be a natural T cell epitope (fig. S13D).

### Probing the energetic landscape of the TCR hotspot by T cell activation and BFP.

We next carried out double-mutant cycle analysis of the 156wt and S32M TCRs with the wt PAP antigen and a PAP-S4A mutant, respectively, to interrogate the functional consequences of this putative interaction suggested from MD and yeast display screening (Fig. 7H, fig. S14). On the PAP peptide chain, Ser4 substitution to Ala boosted activation of 156wt TCR but reduced activation of TCR S32Mα (Fig. 7H, fig. S14). These differences are supported by BFP measurements showing that S4A results in longer bond lifetimes for wt TCR 156 but shorter bond lifetimes for S32M (Fig. 7I and J). The differential sensing of PAP-P4 by 156wt versus S32M TCRs suggests that the TCR hotspot induces a local remodeling that results in an enhanced focus on the PAP-Ser4 side chain that likely manifests as a catch bond.

## Discussion

By leveraging the natural mechanism of TCR activation, which evolved to ensure sensitivity and fine specificity despite low affinity, we used catch bond engineering to overcome the barrier of central tolerance on a T cell specific for a TAA. While affinity maturation of TCR-T can also enhance anti-tumor activities, catch bond engineering offers an alternative mechanism that reduces the propensity for off-target cross-reactivity and maintains the serial triggering mechanism of the TCR because of the preserved low affinity and fast on- and off-rates. The TCR-pMHC off-rate, which reflects the dwell time of the interaction, also correlates with functional parameters, but is not predictive of catch bonds under applied force. This highlights the fundamental difference between catch bond engineering by an activation-based selection that preserves fast off-rate, versus traditional affinity maturation of TCRs through direct off-rate reduction.

The catch bond engineering workflow we presented can be applied to any TCR in the absence of structural information. However, other TCRs will have to be tested empirically on a case-by-case basis, as some may not identify localized catch bond hotspots. Employing three polar amino acids (His/Asn/Glu) with a propensity to form hydrogen bonds or salt bridges, we screened for catch bond hotspots on the TCR through a scanning approach and converged on two amino acids (Ser30 and Ser32) on the CDR1 loop of the TCRα chain. Ser32α seems to be the most critical, since replacing the serine with polar amino acids, namely, glutamic acid, histidine and glutamine, elevate the TCR potency incrementally. Interestingly, methionine, which is a non-polar amino acid, boosted the potency of TCR156 even further. Previous reports suggest that methionine has the potential of forming hydrogen bonds, for example, with the neighboring –NH group of the backbone amino acids (60,61).

We further stress-tested the predictive power of the TCR catch bond regarding *in vivo* T cell efficacy, which had not been established in previous studies. The TCR-PAP/HLA-A2 bond lifetime under force correlates well with the cytokine release, production of cytotoxic molecules by T cells, and it associates positively with the proliferation potencies of the T cells upon antigen stimulation. Supporting these findings, the bond lifetime measured by BFP successfully predicted the tumor cell killing efficacy of the TCR156 mutant T cells, both *in vitro* and in an NSG tumor model. The 3D affinity showed less correlation with TCR potency. For example, TCR S32Qα has similar 3D affinity compared to TCR S32Mα and S30E32Qα, but it could not eliminate PAP^+^ tumor cells as efficiently as the other two TCRs.

The S32Mα and S30E32Qα TCRs delayed the outgrowth of PAP^+^ prostate cancer in the NSG mouse model, suggesting potential applications for adoptive T cell therapy. It is important to note that the NSG mice represent a challenging tumor model for assessing TCR potency due to factors such as MHC mismatch. The transferred human T cells could not function on the tumor stroma in NSG mice via cross-presentation of the PAP antigen, which is critical for tumor rejection in some cases (62-64). Furthermore, the human IFN-γ secreted by the transferred T cells is unable to act on the mouse stroma cells, posing another limiting factor for the model (65-66). Analysis of the TILs indicated that the catch bond engineering endowed the T cells with the capacity to respond to tumor cells *in vivo* whilst the wild-type T cells fail to expand and differentiate into effectors capable of eliminating the tumor. Elongation of the bond lifetime first enhances the effector function of intratumoral T cells, whereas further bond lifetime elongation augments their proliferative capacity in an additive manner. Our results highlight the critical role of T-cell-intrinsic optimal TCR signaling for their survival, proliferation, and effector function in a harsh environment such as tumor microenvironment in NSG mice.

## Supplementary Material

Supplementary_Movie_1_hbonds

Supplementary_Movie_2_hbonds

adx3162_SupplementalMaterial_v6

Materials and Methods

Supplementary Text

Figs. S1 to S14

Table S1

References (67-99)

Supplementary Movies 1 and 2
