# Supplementary material for: Overcoming T cell tolerance to tumor self-antigens through catch-bond engineering
Source: Science. Author manuscript; Available in PMC 2026 Mar 20. (PMC13004167; doi:10.1126/science.adx3162)
Supplement: adx3162_SupplementalMaterial_v6 [file NIHMS2137487-supplement-adx3162_SupplementalMaterial_v6.pdf]

Supplementary Materials for

Overcoming T cell tolerance to tumor self-antigens through catch-bond engineering

Xiaojing Chen, Zhiyuan Mao, E. Motunrayo Kolawole, Margherita Persechino, Kevin M. Jude, Masato Ogishi, Kelvin C. Mo, Jami McLaughlin, Donghui Cheng, Xinyu Xiang, Xinbo Yang, Caitlin Gee, Shiqin Liu, Aerin Yang, Matthias Obenaus, Nan Wang, Miyako Noguchi, Tanya Stoyanova, John K. Lee, Zinaida Good, Naomi R. Latorraca, Brian D. Evavold, Owen N. Witte, K. Christopher Garcia

Corresponding author: OwenWitte@mednet.ucla.edu, kcgarcia@stanford.edu

The PDF file includes: Materials and Methods

Supplementary Text

Figs. S1 to S14

Table S1

References (67-99)

Other Supplementary Material for this manuscript includes the following:

Supplementary Movies 1 and 2
MDAR Reproducibility Checklist

Materials and Methods

Cell lines.

Cells were originally acquired from ATCC. SKW-3 cells, T2 cells, PC3 cells, K562 were kept in RPMI1640+GluMax medium supplemented with 10% FBS, 10 mM HEPES and antibiotics.

PlatE, 293 T cells, LentiX cells were kept in DMEM+GluMax medium supplemented with 10% FBS, 10 mM HEPES and antibiotics.

Lenti-viral production.

LentiX cells were seeded onto 6 well plates at cell density of 0.3×10^6 cell/well. The next day, 0.5 µg psPAX, 0.26 µg pMD2.G and 0.75 µg gene of interest cloned into pHR vector were mixed with 4.5 µL Fugene transfection reagent (Promega) in 100 µl Opti-MEM for 15 min at room temperature and added to the LentiX cells. The transfected LentiX cells were cultured for 48 hours before the viral supernatant was collected and filtered through a 0.45 µm filter (Thermo Fisher Scientific).

Cell line transduction.

All the cell lines were transduced using the lenti-viral transduction protocol. Unless stated otherwise, 10^6 cells were transduced with 1ml of filtered lenti-viral supernatant and 10 µg/ml of polybrene (Millipore, Sigma) and cultured for 5 days before use.

Generation and expression of TCR156 point mutation libraries.

The DNA encoding TCR156α or β chains with N/H/E mutations were synthesized commercially by Twist Bioscience and cloned into pHR lentiviral vector by Gibson assembly. Individual position library was packaged into lentivirus and co-infect SKW-3 reporter cells with the wild-type counterpart TCR chain for at titrated amount to achieve a transduction rate of less than 20% to keep a low MOI.

Generation and expression of DNA shuffled library.

The DNA shuffling was performed as described previously (*67*). In brief, the linear DNAs from the position mutation libraries from either TCR156α or β were generated by enzymatic digestion with *Asc*I and *Sbf*I. The DNAs were then mixed at equal moles. 2µg of the mix DNAs were treated 0.5U of DNase I (Thermo Fisher Scientific) for 3 min at 15°C. The interaction was stopped by adding 50nM EDTA to the interaction and incubated at 65°C for 10 min. The digested DNA was analyzed on an agarose gel and the digested DNA fragments were recovered by gel purification (Qiagen). 200ng of the DNA fragments were assembled using the progressive hybridization protocol using Platinum Tag polymerase (Thermo Fisher Scientific) and the following program: 94°C for 2 minutes; 35 cycles of 94°C for 30 seconds, 65°C for 90 seconds, 62°C for 90 seconds, 59°C for 90 seconds, 56°C for 90 seconds, 53°C for 90 seconds, 50°C for 90 seconds, 47°C for 90 seconds, 44°C for 90 seconds, 41°C for 90 seconds, 68°C for 90 seconds; and the final step of 68°C for 2 minutes. The reaction was cleanup using PCR cleanup kit (Qiagen). The purified assembled DNA was amplified by PCR using the forward primer: 5'-ggagctctcgagaattctcacg-3' and reverse primer: 5'- tgcaggtcgactctagagtcgc-3' and the program: 98°C for 30 seconds; 20 cycles of 98°C for 15 seconds, 60°C for 30 seconds, 72°C for 20 seconds; and the final step of 72°C for

10 minutes. The PCR product was verified on an agarose gel and the correct band was excised and purified using the Qiagen gel purification kit. The shuffled libraries were then cloned into the linearized pHR vector using HiFi assembly kit (New England Biolabs) and transformed into electrocompetent cells MegaX DH10B™ T1R Electrocomp™ Cells (Thermo Fisher Scientific) with the provided protocol: 100 ng of the assembled libraries were incubated with 50 µl of the competent cells on ice for 30 minutes and transferred into a chilled 0.1 cm electroporation cuvette (Bio-rad). The electroporation was conducted at 2.0 kV, 200 Ω, 25 µF with a MicroPulser Electroporator (Bio-rad). The transformed E. *coli* was recovered in 1 ml of S.O.C. medium (Thermo Fisher Scientific) at 37°C for 1 hour. Diluted amount (1:1000 or 1:10 000) of the E. *coli* was plated onto a LB plate supplemented with 100 µg/ml Carbenicillin (Sigma-Aldrich) to determine the colony forming unit (c.f.u.). The rest of the transformed E. *coli* was plated onto a square bioassay dish with LB supplemented with Carbenicillin and grew for 16 hours at 37°C. The colonies were scraped off and used to extract plasmid DNAs that contained the libraries using a Qiagen Plasmid Plus Midi kit (Qiagen). The extracted libraries were packaged into the Lenti-viral vector using the Lenti-X cells as described previously. For TCRα library, TCR156β wild-type chain was co-transfected, and vice versa. The 48 hours viral supernatant was harvested, titrated amount of viral supernatant was used to transduce SKW-3 cells to determine the viral titer to reach a transduction rate of lower than 20%. $3x10^7$ SKW-3 cell libraries were generated for both TCR156α and β and rested for 5 days before library selection.

DNA shuffled library selection.

$1x10^8$ of the SKW-3 cells expressing the initial TCR156α or β libraries were co-cultured with $1x10^8$ T2 cells pulsed with 10^{-7} M of PAP_{22} peptide for 14 hours at 37°C. The cells were harvested and stained for activation marker CD69 using an anti-CD69 antibody (clone FN50, BioLegend) and tetramer PAP/HLA-A2. $CD69^{hi}$ and $tetramer^{low}$ cells were sorted using a SONY SH800S sorter (SONY). The selected cells were rested and expanded for at least 2 weeks before the next selection.

Deep sequencing of the DNA shuffled library.

The beginning and the fifth round of the TCRα libraries were subjected to Miseq deep sequencing system (Illumina) for sequencing. The gDNAs from $1x10^7$ library cells were extracted (QIAGEN). 50 ng of gDNAs were used for PCR amplification with the primer pairs: forward primer of 5'-CTACACGACGCTCTTCCGATCTNNNNNNNN-six nucleotide barcode for different libraries-GATAGCAAGCC-3' and reverse primer of 5'-ATTCCTGCTGAACCGCTCTTCCGATCTNNNNNNNNATTCGGTTTACAACGAGCTGCG-3' and the following program: 98°C for 2 minutes; 30 cycles of 98°C for 10 seconds, 55°C for 30 seconds, 72°C for 20 seconds; and the final step of 72°C for 10 minutes. The PCR product was analyzed on an agarose gel and the correct band was excised and purified (QIAGEN). The adaptor sequence was integrated into the product by a second PCR with the primer pairs: 5'-AATGATACGGCGACCACCGAGATCTACACTCTTTCCCTACACGACGCTCTTCCGA-3' and 5'-CAAGCAGAAGACGGCATACGAGATCGGTCTCGGCATTCCTGCTGAACCGCTCTT C-3', and the program 98°C for 2 minutes; 20 cycles of 98°C for 10 seconds, 60°C for 30 seconds, 72°C for 20 seconds; and the final step of 72°C for 10 minutes. The correct band was purified from a gel and the amount and quality of the PCR product was analyzed using an Agilent Bioanalyzer. The PCR products from different libraries were pooled together at equal mole and diluted to a final

concentration of 8 nM and sequenced using the Miseq Reagent kit v3 (Illumina) with the MiSeq system (Illumina).

Retro-viral production.

Plat-E cells were seeded onto 6 well plates at cell density of $0.3x10^6$ cells/well. The next day, the medium of the Plat-E cells was replaced by 2 ml of fresh, pre-warmed cDMEM medium. 0.375 µg of packaging vector RD114 and 1.125 µg gene of interest cloned into pMSGV-NGFR vector were mixed with 4.5 µL Fugene transfection reagent in 100 µl Opti-MEM for 15 min at room temperature and added to the platE cells. 12 hours later, the medium of the transfected Plat-E cells were replaced by fresh, pre-warmed cRPMI medium. The viral supernatant was collected at time point 24 h and 48 h after medium exchange and filtered through a 0.45 µm filter.

Human primary T cell activation and transduction.

Human whole blood from different human donors were obtained from Stanford Blood Bank under the approved protocol of APB-2749-KG1018. The peripheral blood mononuclear cells (PBMCs) were isolated using Ficoll-Paque (Cytiva). In brief, the human blood was diluted 1:1 with PBS and gently pulled into a Sepmate-50 column (STEMCELL technologies) prefilled with 15ml Ficoll and centrifuged for 20 min without break. The PBMCs were suspended in cRPMI with 300U/ml rIL2 (Peprotech) and plated onto the human CD3 (clone OKT3, biolegend) and human CD28 antibody (clone S20013F, biolegend) pre-coated plate for 48 hours before retro-viral transduction. 1ml of the filtered 24 h viral supernatant was added to PBMCs with 1 µg/ml Protamine Sulfate and centrifuged for 90 minutes at 2000 rpm at 32^oC. The second transduction was conducted as followed: a well of a non-tissue treated 6 well plate was coated with 25ug/ml Retronectin (Takara), 1 ml of 48 h viral supernatant was added to the well and centrifuged at 4000 rpm at 4^oC for 90 minutes. The viral supernatant was discarded and the one-time transduced PBMCs were added to the wells and centrifuged for an additional 30 minutes at 32^oC at 2000 rpm. The PBMCs were kept at 300U/ml rIL2 for 10 days and then 30U/ml rIL2 for another 3 days before use.

Degranulation, Granzyme B, IFNγ and TNFα assay.

$5x10^4$ PAP_{22} peptide pulsed T2 cells or PC3-PAP-A2 cells were co-cultured with $5x10^4$ transduced CD8 T cells. To measure CD107α surface expression, 1x BD GolgiStop protein transport inhibitor (BD Biosciences) was added 1 hour after co-culture. The cells were cultured for another 3 hours and surface CD107α was stained (antibody clone H4A3, Biolegend) together with CD8 (antibody clone SK1, Biolegend) and NGFR (antibody clone ME20.4, Biolegend). For Granzyme B, IFNγ and TNFα, the cells were cultured for 2 hours before 1:1000 GolgiPlug was added, the cells were cultured for another 12 hours. The surface of the cells were stained with CD8 and NGFR and fixed using the BD Cytofix/Cytoperm™ Fixation/Permeabilization Kit and the protocol provided by the manufacturer (BD Biosciences). The intracellular Granzyme B (antibody clone QA16A02, Biolegend), IFNγ (antibody clone MD-1, Biolegend) and TNFα (antibody clone MAb11, Biolegend) were then stained. The cells were then analyzed using a CytoFLEX flow cytometer (Beckman Coulter) and the data was analyzed in Flowjo v10.8.1.

Proliferation assay.

The transduced PBMCs were labeled with 2µM CellTrace Violet according to the provided protocol (ThermoFisher Scientific). $5x10^4$ PC3-PAP-A2 cells were co-cultured with $5x10^4$ labeled

transduced CD8 T cells for 72 hours on a 96-well flat bottom plate, the surface markers CD8 and NGFR were stained before FACs analysis. The percentage of starting culture that underwent proliferation was calculated in Flowjo v10.8.1. The Proliferation Index and Division Index were calculated using "Proliferation modeling" function. The percentage of divided cells was calculated as Divided cells% = Proliferation Index/Division Index.

T cell exhaustion assay.

$2x10^4$ PC3-PAP-A2 cells were added to the culture of $4x10^4$ transduced CD8 T cells every 48 hours for 8 days. The second day after adding of the last batch of PC3-PAP-A2 cells, inhibitory receptors PD-1 (antibody clone EH12.2H7, Biolegend), Tim-3 (clone A18087E, Biolegend), LAG-3 (clone 11C3C65, Biolegend) and TIGIT (clone A15153G, Biolegend) were stained with CD8 and NGFR and analyzed with a CytoFLEX.

For TOX staining, the cells were surface stained for CD8 and NGFR before fixed and permeabilized using eBioscience™ Foxp3/Transcription Factor Fixation/Permeabilization Concentrate and Diluent (ThermoFisher Scientific). The cells were then stained for intracellular TOX expression (clone 6E6D03, Biolegend) and analyzed with a CytoFLEX.

For IFNγ and TNFα staining, the assay was performed as described in the previous section.

***In vitro* Cytotoxicity Measurement Using Incucyte.**

PC3 cells engineered to express both HLA-A2 and PAP (PC3-PAP-A2), were harvested from cultures, resuspended in F-12K media (Thermo Fisher) supplemented with 10% Fetal Bovine Serum (FBS) and glutamine. Approximately 20,000 PC3-PAP-A2 cells were plated into each well of tissue culture-treated 96-well plates (Fisher Scientific). TCR-transduced human peripheral blood mononuclear cells (PBMCs) were collected, washed once, and resuspended in F-12K media supplemented with 10% FBS and glutamine. The numbers of PBMCs were adjusted to the desired concentrations to achieve specific effector to target (E:T) ratios (e.g., 40,000 transduced PBMCs to 20,000 PC3-PAP-A2 for E:T = 2:1; 10,000 transduced PBMCs to 20,000 PC3-PAP-A2 for E:T = 1:2). Each experimental setup was replicated in triplicate, with a total volume of 200 μL per well. The plates were then incubated and analyzed using the Incucyte S3 live-cell analysis system (Sartorius) at 37°C. The total area of green objects (contributed by green fluorescent protein or GFP) was recorded every two hours by capturing four images per well to monitor the proliferation of GFP-positive PC3-A2-PAP cells. For repetitive tumor cell challenge assays, 100 μL of culture supernatant was removed every 48 hours and replaced with fresh F-12K media containing an additional 20,000 PC3-PAP-A2 cells in 100 μL, supplemented with FBS and glutamine. For both in vitro and in vivo cytotoxicity studies, PBMCs from at least three individual healthy donors were retrieved, processed, and evaluated.

***In vivo* tumor killing using PC3 xenografts.**

Human peripheral blood mononuclear cells (PBMCs) used for the in vivo tumor killing assay were infected and cultured following previously described methods, with slight modifications (*68*). On day 0, cryopreserved PBMCs obtained from healthy donors (AllCells) were activated overnight using CD3/CD28 dynabeads (Thermo Fisher) at a ratio of 25 μL beads per million PBMCs. For experiments using CD4 and CD8 T cells separately, CD8 and CD4 positive selection kits (Miltenyi Biotec) were used prior to adding dynabeads. The PBMCs and dynabeads were resuspended at one

million PBMCs per mL in T cell media (TCM), which included AIM V media (Thermo Fisher), 5% Human AB serum (Omega Scientific), 50 U/mL human recombinant IL-2 (Peprotech), 1 ng/mL human recombinant IL-15 (Peprotech), 1X Glutamax (Thermo Fisher), and 50 μM β-mercaptoethanol. Each 2 mL cell and dynabead mixture was then plated in a cell culture-treated 24-well plate per well and incubated at 37°C. After overnight activation (day 1), 1.5 mL of the cell culture supernatant was removed and replaced with 2 mL of retroviral supernatant containing 10 μg/mL polybrene. For spin infection, plates were centrifuged at 1,350 x g for 90 minutes at 30°C. This procedure was repeated the following day (day 2). On day 3, PBMCs were washed once with 1X phosphate-buffered saline (PBS) without calcium and magnesium to remove residual viral particles and resuspended in fresh TCM. On day 5, CD3/CD28 dynabeads were removed, and the PBMCs were counted and resuspended in fresh TCM at one million per mL. Flow cytometry was conducted to analyze cell phenotypes (CD3, CD8) and assess the transduction (LNGFR) and membrane trafficking of the target TCRs (murine TCRβ). On day 7, transduced PBMCs were harvested, washed twice with 1X PBS, and resuspended at a concentration of 5 x 10^6 cells per 100 μL of 1X PBS for injection into animals.

Immunodeficient NOD scid gamma (NSG) mice aged 8-12 weeks were acquired from The Jackson Laboratory. PC3-A2-PAP cells were harvested, washed twice, and resuspended in 1X PBS at a concentration of 10^7 cells/mL. One million PC3-PAP-A2 cells in 100 μL of 1X PBS were then injected subcutaneously into the right flank of the NSG mice to establish tumor xenografts. The xenografts were allowed to grow for one week prior to PBMC injection, which was performed either intratumorally or intravenously (via the tail vein). One week post-injection of intravenous delivery, retro-orbital bleeding was performed, and flow cytometry was utilized to confirm the circulation of transduced PBMCs. Tumor growth was monitored weekly using calipers. Animals were euthanized eight weeks after PBMC injection, and tumors and organs were harvested for analysis. All procedures were conducted in accordance with protocols approved by the University of California, Los Angeles (UCLA) Division of Laboratory and Animal Medicine (DLAM) and UCLA Chancellor's Animal Research Committee (ARC) under ARC-2005-068. Sample size was determined using the resource equation approach in the context of a one-way ANOVA comparison. Specifically, the minimum number of animals required was calculated using the formula $n=DF/k+1$, where DF is the minimum degrees of freedom (assumed to be 10), and k is the number of groups (three: T cells transduced with an irrelevant TCR, wild-type TCR156, and a catch bond-engineered TCR). This calculation yielded approximately 4.3 animals per group, which was then rounded up to 5. Therefore, five animals were used per group. To minimize bias, the researchers measuring tumor sizes were blinded to the treatment cohorts.

Immunohistochemistry (IHC) staining.

Tumor xenografts were fixed in 10% formalin overnight at 4 °C and transferred to 70% ethanol before processing. The tumors were processed sequentially through 70% ethanol, 95% ethanol with 5% methanol, 100% ethanol, Clearify, and heated paraffin. After processing, the tumors were embedded in paraffin, and paraffin-embedded sections were cut into 4 μm slices. The slides were heated at 65 °C for one hour, deparaffinized using Clearify, and rehydrated through a graded ethanol series (100%, 95%, and 70%). For antigen retrieval, the slides were incubated in 10 mM sodium citrate buffer (pH 6.0) at 95 °C for 30 minutes. Endogenous peroxidase activity was inhibited by incubating the tissue sections with 3% hydrogen peroxide for 5 minutes. The slides were then blocked with 2.5% goat serum for 1 hour at room temperature. After blocking, the slides were incubated overnight at 4 °C in a humidified chamber with the following primary antibodies:

anti-HLA Class 1 ABC antibody (EMR8-5, Abcam), anti-CD3ε antibody (Cell Signaling Technology), or anti-ACPP antibody (HPA063916, Sigma). Following three washes with 1x PBS, the slides were incubated with horseradish peroxidase (HRP)-conjugated secondary antibodies (Goat Anti-Mouse/Rabbit IgG Polymer Detection Kit, Peroxidase, Vector Laboratories) for 1 hour at room temperature. The sections were washed three times with 1x PBS, developed using DAB (DAKO), and rinsed with water. The sections were stained with hematoxylin, dehydrated through graded ethanol (70%, 95%, and 100%), mounted, and imaged using a histology microscope.

Western blot analysis.

Cell lines were lysed using Urea lysis buffer (8M Urea, 4% CHAPS, 2X protease inhibitor cocktail (Roche/Sigma Aldrich #11697498001) to extract proteins. Mixtures were then sonicated and ultracentrifuged using Beckman Optima MAX-XP for 90 minutes at 45,000 RPM to remove genomic DNA. Protein concentrations were measured using BCA assays (Pierce, #23225). Invitrogen iBright 1500 was used to visualize the results. Antibodies used: anti-N4BP2 antibody (23892-1-AP, Proteintech, dilution = 1:2,000), anti-GAPDH antibody conjugated with HRP (GTX627408-01, GeneTex, dilution = 1:5,000), and goat anti-rabbit IgG (H+L)-HRP conjugate (Bio-Rad, # 1706515).

Tumor infiltrating lymphocytes harvesting and single-cell sequencing.

Similar to previously mentioned tumor models, one million PC3-A2-PAP cells in 100 µL of 1X PBS were injected subcutaneously into the right flank of NSG mice to establish tumor xenografts. Six weeks after tumor engraftment, 5 million TCR-engineered PBMCs were administered intravenously via the tail vein. Five animals were included in each group. Three out of five animals with similar sizes of tumors were sacrificed 10 days post-injection. Tumors were excised, cut into 2-3 mm pieces, and digested with 1 mg/mL Collagenase Type I (Gibco) and 1 mg/mL Dispase (Gibco), supplemented with Rock inhibitor (Tocris Bioscience) and DNase I (Roche, Sigma), at 37°C for one hour. This was followed by a 5-minute treatment with TrypLE (Gibco). The processed cells were then filtered through a 40 µm filter and stained with anti-human CD3 (clone SK7, eBioscience), NGFR (Biolegend), and anti-mouse CD45 (clone 30-F11, eBioscience). Cells identified as DAPI-muCD45-$CD3^{+}NGFR^{+}$ were single-cell sorted into 1X PBS with 0.04% bovine serum albumin (BSA). Cells from the same group (n = 3) were pooled and subsequently processed by the UCLA Technology Center for Genomics and Bioinformatics (TCGB) core for 10X Genomics GEM-X 3’ Gene Expression v4 library preparation and next-generation sequencing on the Illumina Novaseq X platform.

sc-RNAseq data analysis.

The FASTQ files were processed by Cell Ranger v9.0.0. The gene expression matrix was processed and analyzed using Seurat (version 5.1.0) (*69-73*). For quality control, we excluded cells that contained less than 500 read counts for genes (minimal cutoff), or more than 100,000 read counts for genes (maximum cutoff) and also cells in which more than 20% of transcripts were derived from mitochondrial RNA, leaving 10,616 cells. Data were log-normalized and scaled using Seurat's default workflow. Uniform Manifold Approximation and Projection (UMAP) embedding in their original publication (*74*) (see their Fig. 2a) was reconstructed, and our data were projected onto the reconstructed UMAP embedding space using the *MapQuery* function implemented in Seurat. Clustering and trajectory inference analysis was performed using Monocle 3 (version 1.3.7)

(*75-77*). Differential expression (DE) analysis between groups was performed via the Wilcoxon's rank sum test implemented in Seurat's *FindMarkers* function. Gene set enrichment analysis (GSEA) was performed using the log2FC ranking of DE genes using fgsea. Human Hallmark gene sets were retrieved from MSigDB. The Effector score was calculated using Seurat's AddModuleScore function, with GZMA, GZMB, GZMH, GNLY, PRF1, and IFNG as an input gene set. To calculate Cell Cycle score, Seurat's CellCycleScoring function was used. Built-in genesets for the S and G2M phases in Seurat were used to calculate S and G2M scores. CellCycle score was defined as the sum of S and G2M scores.

Refolding of PAP/HLA-A2.

Refolding of PAP/HLA-A2 was performed as described previously (*36, 78*). Refolding buffer was prepared as 100 mM Tris-HCl pH 8.0, 400 mM L-Arginin, 5 M Urea, 0.5 mM oxidized glutathione, 5 mM reduced glutathione, 2 mM EDTA. 26 mg PAP was dissolved in DMSO and added to 500 ml refolding buffer. 28 mg HLA-A2 inclusion body and 28 mg human β-2-microglobulin inclusion body were mixed and added into the refolding buffer drop by drop. Then, the refold buffer/protein were poured into dialysis tubing (Spectrapor RC 10 kDa MW cutoff) and dialyzed against 5 L 10 mM Tris.Cl, pH 8.0 for 5 days, with the Tris buffer changed every 24 hours. The protein was filtered, concentrated to 500 µL and biotinylated overnight. Biotinylated refolded protein was purified by size exclusion chromatography (Superdex 200, GE Healthcare) and ion exchange (MonoQ, GE Healthcare) on AKTA Purifier (GE Healthcare).

Production of TCR protein in Expi293/Expi293F GnTI- cells.

The TCR protein used for SPR was produced in Expi293 cells and the proteins for crystallography was produced in Expi293F GnTI- cells (Thermo Fisher Scientific). Specifically, TCR156α chain was cloned into pD649 vector with basic zipper and 6xHis tag, and TCRβ chain was cloned into pD649 vector with acid zipper and 6xHis tag. 1 µg TCRα and 1 µg TCRβ construct were transfected into 10^6 Expi293 GnTI- cells according to the manufacturer's protocol. The supernatant of the transfected Expi293 GnTI- cells was harvested 4 days later, diluted with equal volume of PBS and 20mM (final concentration) Tris.Cl (pH 8.0). The supernatant was incubated with 2ml Ni-NTA (Thermo Fisher Scientific) at 4°C overnight, the Ni-NTA was washed twice with 10 mM Imidazole and the bound protein was eluted using 300 mM Imidazole. The TCR protein was buffer-exchanged with HBS and concentrated with a 30 kDa filter (Millipore). The TCR proteins for crystallography were then treated with Caboxypeptidase A/B, EndoH and 3C protease to remove sugars and C' zippers at 4oC overnight. The protein was purified by size-exclusion chromatography using Superdex200 column on AKTAPurifier (GE Healthcare) and the size was confirmed using a SDS-Page gel.

BFP measurements.

The BFP force clamp assay has been described (*42*). In brief, SKW-3 cells expressing TCR156 variants were aspirated onto a piezo driven micropipette controlled by Labview (National Instrument) programs. An opposing micropipette as an aspirated RBC biotinylated with EZ-link NHS-PEG-Biotin (Thermo Fisher Scientific). At the apex of this RBC was a streptavidin-maleimide (Sigma-Aldrich) bound glass bead coated with the PAP-HLA-A2. This RBC:bead complex served as a force probe sensor. The SKW-3 cell was repetitively brought into contact with the RBC, held and then retracted to the distance controlled by the piezo actuator. The

retraction and hold phase generated a force on the TCR:MHC bond, which could be altered, based on the distance the T cell was retracted. The position of the edge of the bead was tracked by the high-resolution camera (1,600 frames/sec) with < 3 nm displacement precision. The camera then recorded the time it took for the T cell to disengage the glass bead, which can visually be seen by the RBC retracting and the bead returning to its starting position. Multiple repeated cycles (known as force-clamp cycles) could be carried at a single force in order to generate an average bond lifetime between the TCR and peptide:MHC complex. Varying the level of force and recording lifetimes allowed for the determination of the average bond lifetime and the type of bond formation.

Surface plasmon resonance measurement.

The affinity of TCR156 variants binding to the PAP/HLA-A2 was measured by surface plasmon resonance on Biacore T100 (GE Healthcare). The PAP/HLA-A2 tetramer was generated by UV-exchange and immobilized on a streptavidin chip (GE Healthcare). The TCR156 proteins produced in Expi293 cells were treated with 3C protease to remove the basic/acid zipper. The pMHC protein was immobilized until an increase of around 200 in RU, and the titrated TCR protein was flowed through the flow cell at 25 °C. The affinity of steady-state was determined by the Biacore T100 evaluation software. For the kinetic fitting, Response Index (RI) was set to be constant to 0.

TCR156-PAP/HLA-A2 crystallography.

Purified TCR156s and refolded PAP/HLA-A2 were mixed at 1:1 molar ratio at 8 mg/ml concentration for crystallization screening. Crystals of wtTCR156-PAP/HLA-A2 and of the S32Mα complex were grown in 0.2 M Sodium malonate, 0.1 M bis Tris-propane pH 7.5, and 17% PEG3500. Crystals of the S32Hα complex were grown in 0.2 M Sodium malonate, 0.1 M bis Tris-propane pH 7.5, and 20% PEG3500. Crystals of the S32Qα complex were grown from 0.2 M sodium malonate and 20% PEG 3350. Initial crystals of the S30E32Qα complex were grown in 0.2 M NaBr, 0.1 M bis tris propane pH 7.5, and 20% PEG 3350, then used as a microseed stock to grow the final crystals in 0.2 M Na_2SO_4, bis tris propane pH 7.5, and 20% PEG 3350. All crystals were snap frozen in mother liquor containing 30% glycerol. Diffraction data were collected at the Advanced Light Source beamlines 2.0.1 (S32Hα) and 8.2.1 (all others). Datasets were indexed and scaled with XDS (*79*), space groups were determined with Pointless, and reflections were merged with Aimless (*80,81*). The structure of the S32Hα complex was solved by molecular replacement (MR) in Phaser (*82*) using HLA from PDB entry 6TRN, separated alpha chain constant and variable domains from PDB entry 7PDX, and separated beta chain constant and variable domains from PDB entry 8CX4 as search models. The remaining structures were solved by MR using the TCR and HLA-peptide from the refined S32Hα complex. The S30E32Qα and wt complexes were initially rebuilt with phenix.autobuild (*83*), and all structures were completed by iterative cycles of rebuilding and refinement using coot (*84*) and phenix (*85-87*). 2mFo-DFc omit maps for each structure were calculated in phenix by setting the PAP peptide occupancy to 0 and subjecting the structure to simulated annealing from 5000 K (Fig. S12C-G). Final refinement parameters included reciprocal space XYZ, TLS, and individual ADP refinement. Model quality statistics were measured using MolProbity (*88*). Data collection and refinement statistics are reported in Supplementary table 1. Crystallographic software used in this project was installed and configured by SBGrid (*89*).

A2 yeast library selection.

The A2 yeast library construction and TCR selection were performed according to previously published method (*90*). In brief, a single chain linking the randomized nonamer peptide library, β2m and HLA-A*02:01 α1, α2 and α3 extracellular domain were inserted to the *N*-terminus of Aga2 on the yeast expressing vector pYAL. The HLA-A*02:01 heavy chain contains Y84A mutation to allow an opening at the terminal of the A2 chain for peptide loading. HA tag was added to the C-terminus of the single chain A2 trimer. The insert encoding the library and the linear vector were electroporated into EBY-100. An A2 library with $8x10^8$ diversity was generated this way. For each round of selection, the library with a cell number that was ten times of the diversity from the last round was cultured in SDCAA, pH 4.5 until the OD reached 3-4. Same starting amount of cultured yeast was then transferred to SGCAA, pH 4.5 until OD of 3-4 for the induction of the A2 single chain construct expression. After 48 hours of induction, the yeast were harvested and incubated with Streptavidin magnetic MicroBeads (Miltenyi, # 130-048-102) for 1 hour to exclude for unspecific binding yeast. Biotinylated TCRs produced in Expi293 cells were then coated onto Streptavidin magnetic MicroBeads to select for TCR binding yeast.

Yeast library deep sequencing.

The plasmids from different rounds of selection were extracted using Zymoprep II Kit (#D2004, Zymo Research). The peptide libraries sequences were amplified using the PCR primers: forward primer of 5'-CTACACGACGCTCTTCCGATCTNNNNNNNN-six nucleotide barcode for different libraries- TGCAATCCGTCGTGTTTTTGC-3' and reverse primer of 5'-ATTCCTGCTGAACCGCTCTTCCGATCTNNNNNNNNTGTATGGACCCACCCCCGC-3' and the same programs as for DNA shuffled library. The PCR products from different libraries were pooled together at equal mole and diluted to a final concentration of 8 nM and sequenced using the Miseq Reagent kit v2 (Illumina) with the MiSeq system (Illumina).

Analysis of the deep sequencing data and prediction of peptides from human proteome that shared similar motifs.

Only the forward reads from the sequencing results were used for following analysis. The peptides were trimmed from the sequences and the frequencies of the peptides were counted by custom Perl Scripts (*90*). Round 3-4 sequencing results from the selection were used to generate position weight matrices (PWM). The PWM from each TCR selection was used to predict wildtype peptides from human proteome from UniProtKB (Proteome ID UP000005640; June 2020 update) (*90*).

Molecular Dynamics simulations.

We initiated simulations of TCR WT and TCR S32Mα with PAP/A2 complexes from the crystal structures determined in this manuscript. To reduce system size for simulation, we removed chains peripheral to the central core of the complex. That is, we retained MHC residues 1–181 (chain A); peptide residues 1–9 (chain C); TCRα residues 1–110 (chain D); and TCRβ residues 1–113. We retained crystallographic waters and glycan modifications. Prime (Schrödinger) was used to model hydrogen atoms and to add neutral acetyl and methylamide groups to cap protein termini, while leaving peptide termini uncapped. In the case of alternative rotamers, we selected to use the default rotamer selected by Schrödinger; for the peptide Ser4 in the S32M structure, we selected the rotamer that contacts Asn92 (TCRα). We retained titratable residues in their dominant protonation state at pH 7.0, resulting in protonation of Asp77 in chain A. By default, we employed neutral

histidine tautomers with protonation on the delta nitrogen, unless protonating the epsilon nitrogen improved the hydrogen bonding network. His3 (chain A) was positively charged.

We used tleap in AmberTools (*91*) to build the simulation box and apply force-field parameters. Box dimensions were chosen to maintain a ~20 Å buffer between the protein image and the edge of the box, resulting in a box size of 121 Å × 118 Å × 119 Å (214,408 atoms; WT) and 122 Å × 117 Å × 122 Å (220,854 atoms; S32M). Sodium and chloride ions were added to neutralize the system to a concentration of 150 mM. We applied the ff19SB protein force field, the four-point OPC water model, and the corresponding 12-6-4 ion force field (*92*, *93*). Atom counts include 'dummy' atoms employed in the four-point OPC water model.

We initiated simulations using the Compute Unified Device Architecture version of Particle Mesh Ewald MD with the AMBER24 software on single graphics processing units (GPUs) (*94*, *95*). Systems were minimized in two stages, each composed of steepest descent minimization followed by conjugate gradient minimization. Systems were then heated from 0 to 100 K in the NVT ensemble over 12.5 ps and then from 100 K to 310 K in the NPT ensemble over 125 ps at 1 bar, with harmonic restraints of 5.0 kcal mol-1 Å-2 placed on all protein atoms and crystallographic water molecules. Systems were subsequently equilibrated at 310 K in the NPT ensemble at 1 bar over 60 ns. Harmonic restraints were tapered by 1.0 kcal mol-1 Å-2 over 30 ns and then from 0.5 kcal mol-1 Å-2 by 0.1 kcal mol-1 Å-2 over 30 additional ns. Production simulations were carried out in the NPT ensemble at 310 K and 1 bar, using a Langevin thermostat for temperature coupling and a Berendsen barostat with isotropic pressure control (*96*). Nonbonded interactions were cut off at 10.0 Å; long-range electrostatic interactions were calculated using Particle Mesh Ewald with an Ewald coefficient of 0.27511 and a B-spline interpolation order of 4. The FFT grid size was chosen such that the width of each grid cell was ~1 Å. We used hydrogen mass repartitioning with a 4-fs time step and constrained bond lengths to hydrogens using SHAKE (*97*). Trajectory snapshots were saved every 200 ps.

Simulation analysis was performed using Visual Molecular Dynamics (VMD) and the MDAnalysis python package (*98*, *99*). Hydrogen bonds were assessed using VMD's built-in hydrogen bond function using a donor–acceptor distance of 3.5 Å and donor–hydrogen–acceptor angle of 60º. To compute waters near the S32 pocket, we identified all water oxygen atoms within a sphere of radius 4.5 Å, centered on the crystallographic M32 sulfur atom after alignment of all simulation frames on chain A (MHC).

Supplementary movie captions:

Supplementary Movie 1. Representative 1-μs simulation of wt156 (simulation 4) reveals polar network coordinated by Ser32 (TCRα), Asn92 (TCRα), the backbone carbonyl of Asn93 (TCRα), Ser4 (peptide), and Gln155 (MHC). Water molecules shown are those within a 4.5 Å bounding sphere centered on the sulfur atom of S32M after alignment of wt156 simulation frames to the S32M crystal structure (see Methods). Protein atoms are smoothed using a moving average over every ten simulation frames (10 ns). TCRα shown in blue, peptide in orange, and MHC in yellow. Hydrogen bonds are identified using PyMOL's built-in hbond function with a donor–acceptor distance cutoff of 3.2 Å.

Supplementary Movie 2. Representative 1-μs simulation of S32M (simulation 1) reveals effect of Met32 (TCRα) on the network involving Asn92 (TCRα), the backbone carbonyl of Asn93 (TCRα), Ser4 (peptide), and Gln155 (MHC). Water molecules shown are those within a 4.5 Å bounding sphere centered on the sulfur atom of S32M after alignment simulation frames to the S32M crystal structure (see Methods). Protein atoms are smoothed using a moving average over every ten simulation frames (10 ns). TCRα shown in pink, peptide in orange, and MHC in yellow. Hydrogen bonds are identified using PyMOL's built-in hbond function with a donor–acceptor distance cutoff of 3.2 Å.

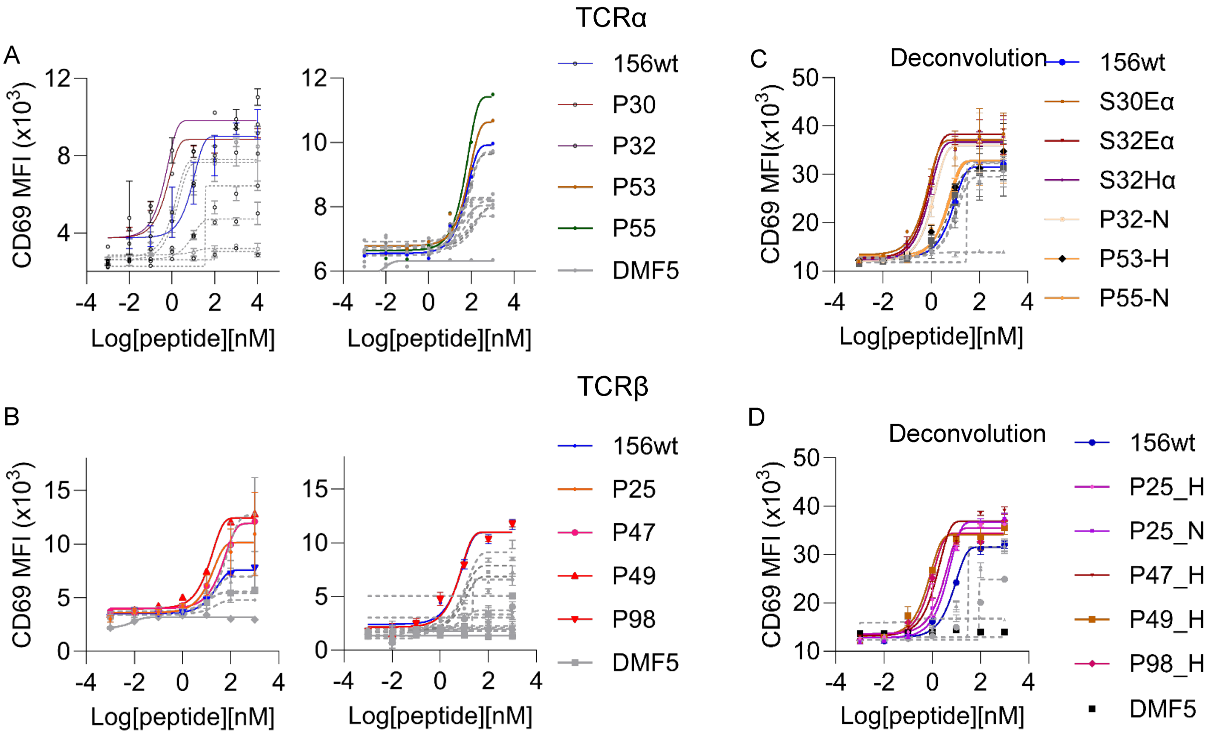

Fig. S1: Position scanning of all positions of TCR156α (**A**) or β chain (**B**). The positions that showed enhanced recognition were further deconvoluted for α (**C**) and β (**D**). All the other positions on the CDRs that did not show enhancement of CD69 expression are shown in grey. The experiments were performed in duplicates and repeated at least once.

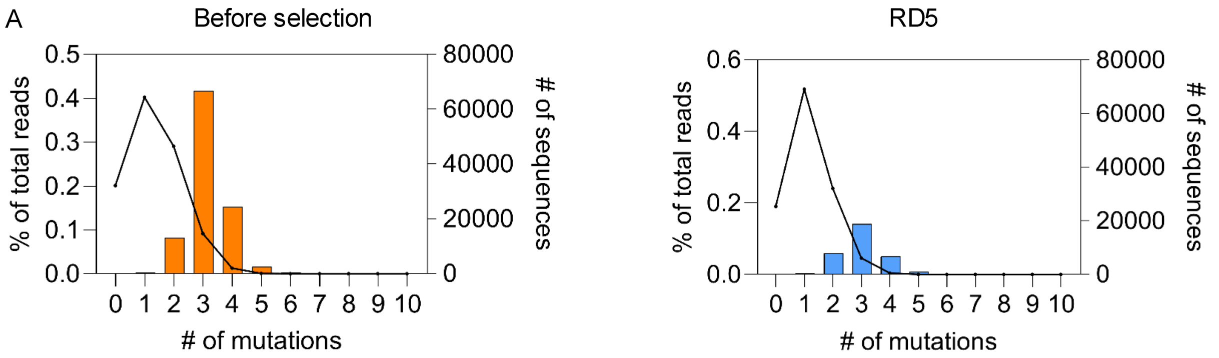

B

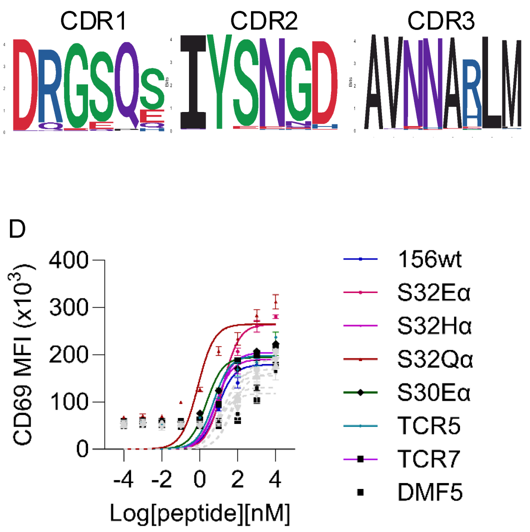

C

TCR clone	CDR1	CDR2	CDR3
**wt**	DRGSQS	IYSNGD	AVNNARLM
**TCR1**	DQGSQS	IYSNGD	AVNNARLM
**S32Eα**	DRGSQE	IYSNGD	AVNNARLM
**TCR2**	DRGSQS	IYSNGD	AVNNAHLM
**S30Eα**	DRGEQS	IYSNGD	AVNNARLM
**S32Qα**	DRGSQQ	IYSNGD	AVNNARLM
**TCR4**	DQGSQS	IYSNGD	AVNNAHLM
**TCR5**	DRGSQS	IYSNND	AVNNARLM
**TCR6**	DRGSQE	IYSNGD	AVNNAHLM
**TCR7**	DRGSQH	IYSNHD	AVNNARLM
**S32Hα**	DRGSQH	IYSNGD	AVNNARLM
**TCR8**	DRGEQS	IYSNGD	AVNNAHLM
**TCR9**	DRGSQQ	IYENGD	AVNNAHLM

Fig. S2: DNA shuffled library.

The beginning library and selected library from round 5 were deep sequenced.

(A) The coded amino acids from random combinations of the genetic codes.

(B) The diversities of the beginning library (left plot) and the selected library at round 5 (right) that contained different number of the mutations on a single chain.

(B) Sequence logo of round 5 selected library on the CDR1, 2 or 3 loop.

(C) Sequences of the wild-type and the top 12 clones selected from the DNA shuffled library.

(D) Functional comparison of the top 12 selected TCR156 variants with the wild-type TCR in a peptide titration assay. The clones that did not show CD69 enhancement are shown in grey. The experiment was performed in duplicates and repeated once.

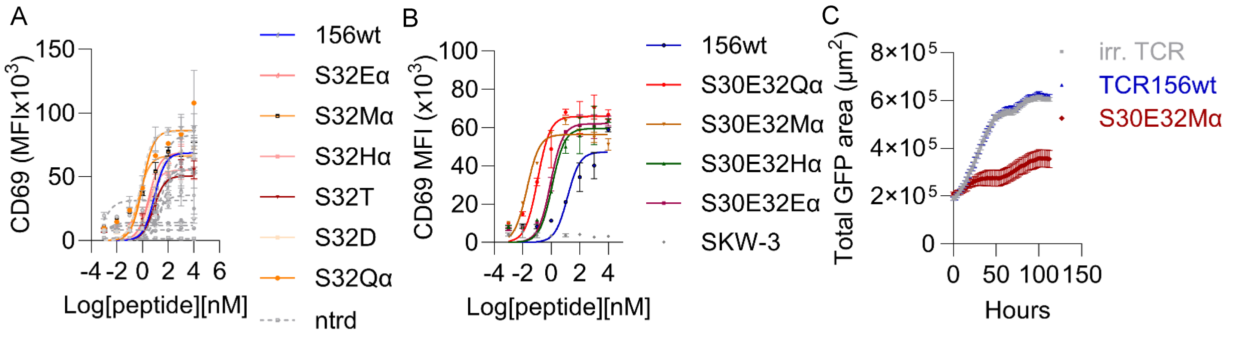

Fig. S3: TCR156 position 30 and 32 mutational scan. (**A**) Ser32 or (**B**) combinatorial mutants of Ser30E and Ser32 were expressed in SKW-3 cells and compared to the wild-type TCR in a titration assay. The experiment was performed in duplicates and repeated once.
(**C**) Incucyte analysis indicating that S30E32Mα inhibited PC3-A2 cell growth compared to the irrelevant TCR and wild type TCR groups. The PC3-A2 was pre-treated with IFNγ for 24 hours.

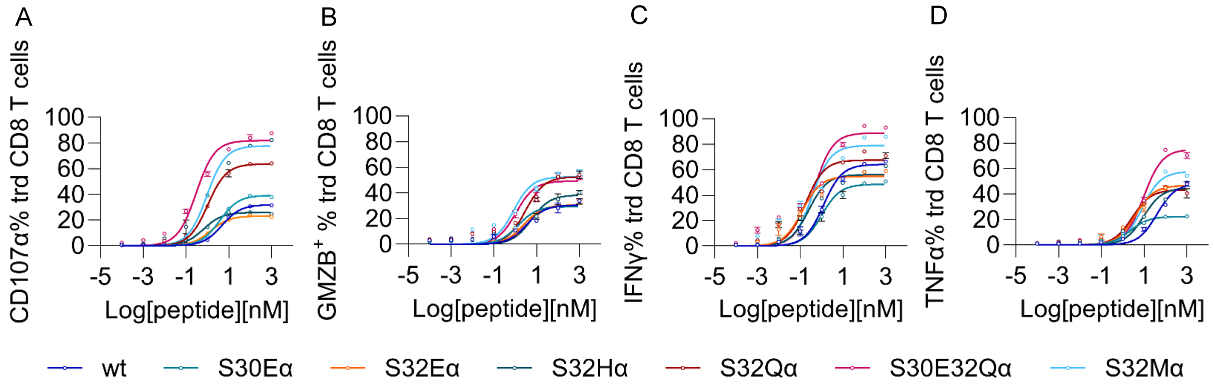

Fig. S4: Peptide titration assay to measure primary T cell response in upregulation of CD107α on T cell membrane (**A**), production of granzyme B (**B**), IFNγ (**C**) and TNFα (**D**).

5

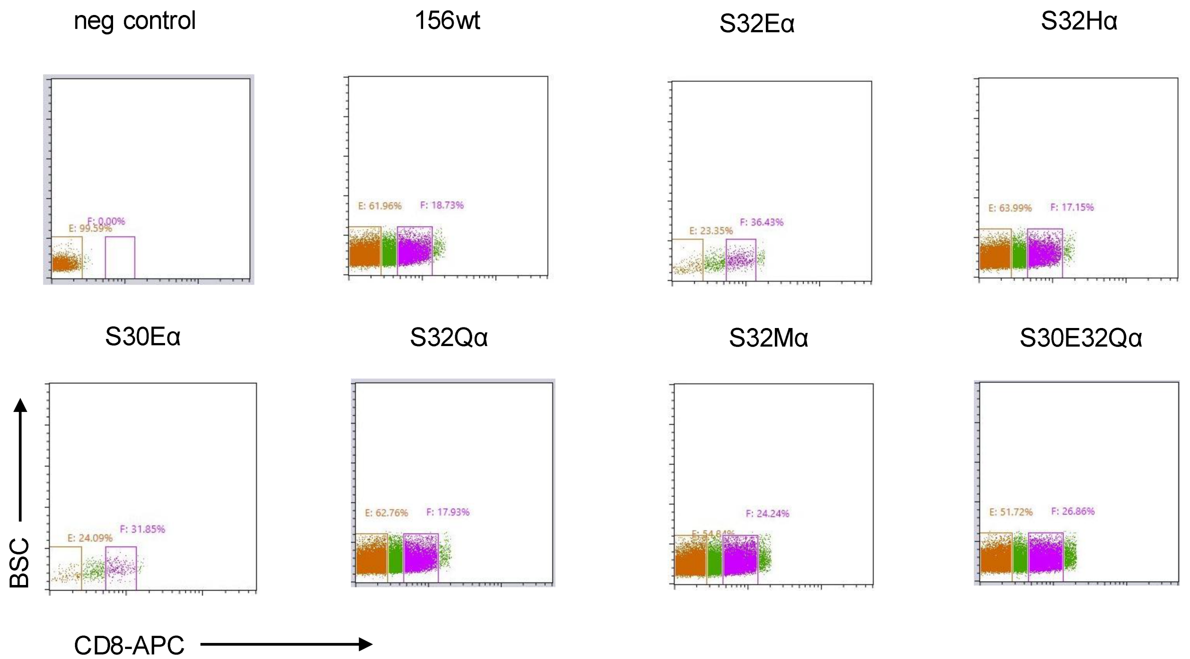

Fig. S5: FACs sort to normalize CD8 expression on SKW-3 T cell expressing different TCR156 variants.

A

	Peak bond life time (sec, BFP)		SPR			
		K_d (μM)	k_{off}/k_{on} (μM)	k_{on} ($M^{-1}sec^{-1}$)	K_{off} (sec^{-1})	$t_{1/2}$ (sec)
156wt	0.19	30	24	20000	0.48	1.45
S32Eα	0.60	40	45	4453	0.20	3.47
S32Hα	0.76	61	98	2100	0.21	3.30
S30Eα	0.97	68	25	8732	0.22	3.15
S32Qα	1.64	9.8	6.1	14640	0.09	7.7
S32Mα	4.96	5.3	2.7	16910	0.046	15.06
S30E32Qα	5.51	11.5	7.8	2573	0.02	34.65

B

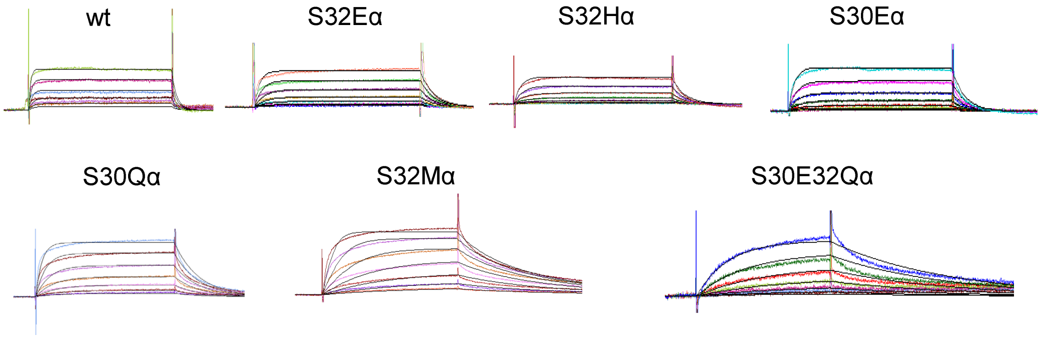

Fig. S6: Table of surface plasmon resonance (SPR) measurements from both equilibrium (K_d) and kinetic measurement methodologies.

(A) Table of K_d, k_{off}, k_{on}, $t_{1/2}$ and K_d calculated from k_{off}/k_{on}

(B) Kinetic sensorgrams of the TCR156 and mutants with PAP22/A2 from kinetic measurements.

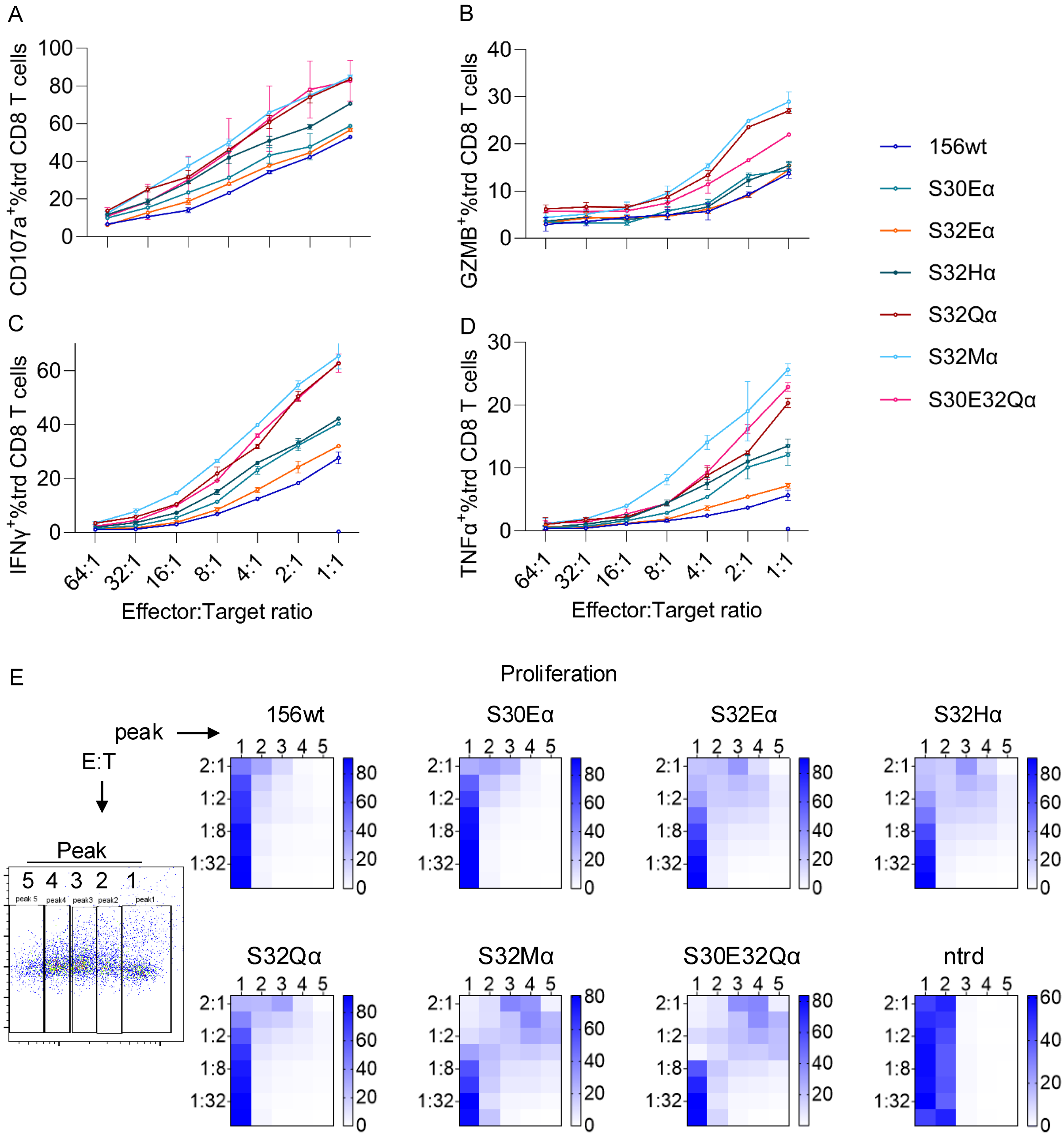

Fig. S7: T cell profiling of the TCR156 mutants.

(**A-D**) The percentage of T cells that showed membrane upregulation of CD107α (**A**), T cell production of granzyme B (**B**), IFNγ (**C**), and TNFα (**D**), of $5x10^4$ human T cells and titrated amount of PC3-PAP-A2 cells were co-cultured to achieve different E:T ratios.

(**E**) Heatmap showing the percentages of TCR156-T cells that divided different times at different E:T ratios.

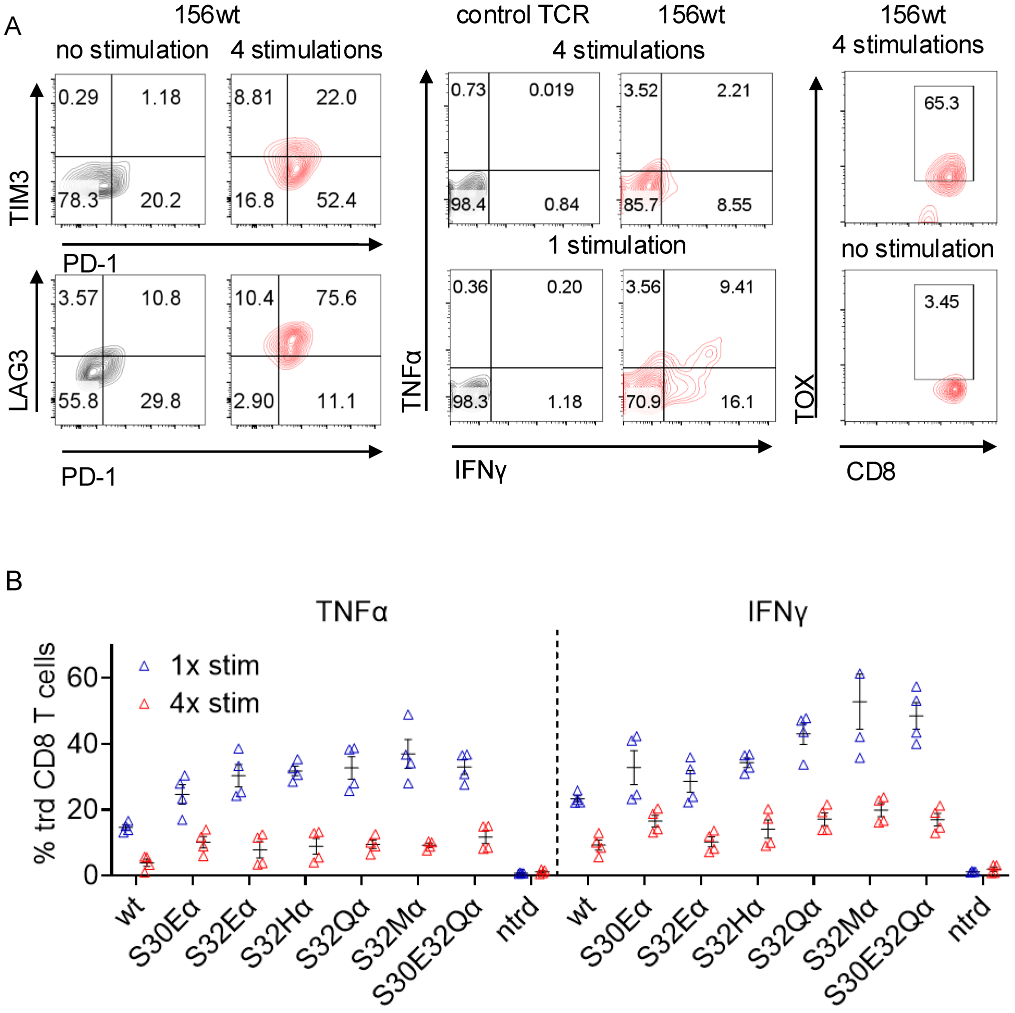

Fig. S8: ***in vitro*** **exhaustion assay with repetitive PAP antigen stimulation.**
(**A**) Exhaustion state of the wild-type TCR156 T cells at day 7-8 after repetitive stimulation assessed by surface IRs (left panels), cytokines TNFα and IFNγ secretion (middle panels) and TOX upregulation (right panels). The exhaustion states were determined on day 7-8. Representative FACs plots from results from 3 donors were shown.

(**B**) The IFNγ and TNFα production of TCR156 T cells after four rounds of antigen stimulation (4x stim) compared to the non-exhausted T cells (1x stim). 12 hours after the last round of PC3-PAP-A2 stimulation, the IFNγ and TNFα production in TCR156 T cells were measured by intracellular staining.

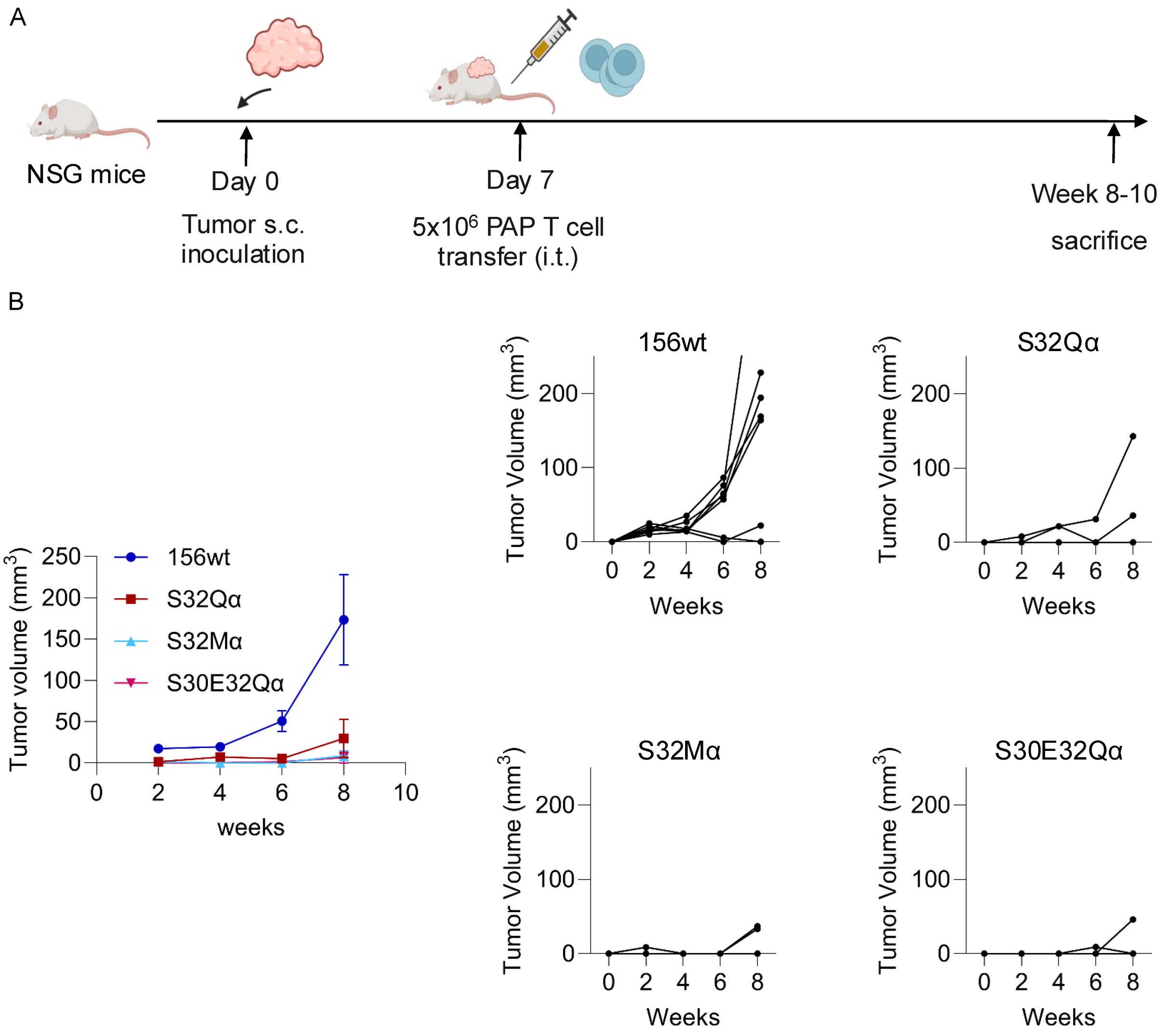

Fig. S9: Adoptive T cell therapy in NSG mice with intratumorally delivered PBMC cells expressing different variants of TCRs.

(A) Timeline of adoptive T cell transfer (intratumoral or i.t.) for tumor treatment in NSG mice.

(B) tumor size measurement of mice treated with different TCR expressing human PBMCs either as average (left panel) or individual mice (right panels).

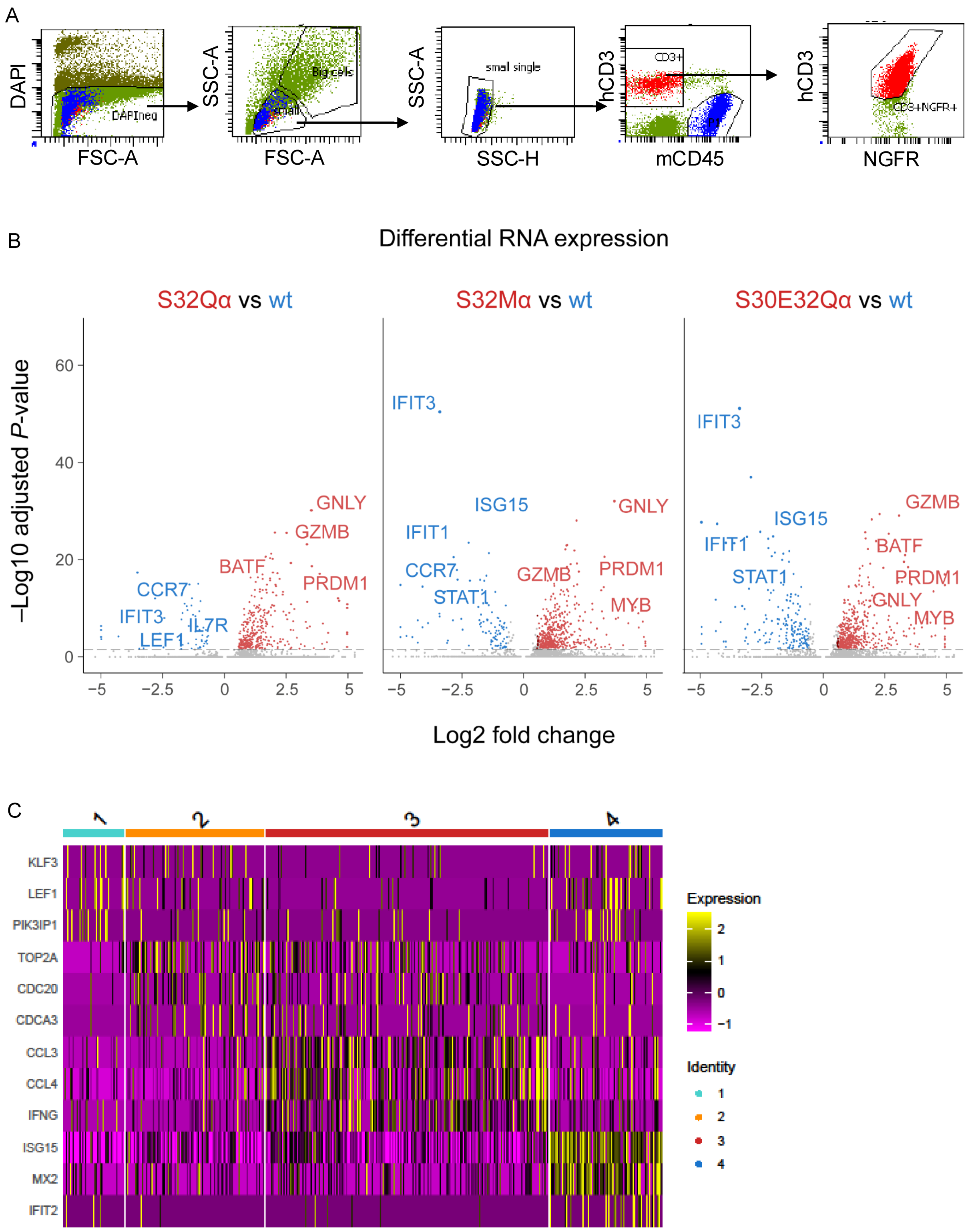
A
DAPI
FSC-A
DAPIneg
SSC-A
FSC-A
Big cells
small
SSC-A
SSC-H
small single
hCD3
mCD45
CD3+
hCD3
NGFR
CD3+NGFR+
B
Differential RNA expression
S32Qα vs wt
S32Mα vs wt
S30E32Qα vs wt
−Log10 adjusted P-value
Log2 fold change
GNLY
GZMB
BATF
PRDM1
CCR7
IFIT3
IL7R
LEF1
ISG15
IFIT1
STAT1
MYB
C
KLF3
LEF1
PIK3IP1
TOP2A
CDC20
CDCA3
CCL3
CCL4
IFNG
ISG15
MX2
IFIT2
Expression
Identity
1
2
3
4

Fig. S10: scRNAseq analysis of the TCR156 TILs.

(A) Sorting strategy for isolating TILs used in scRNAseq with S32Mα sorting as an example.

(B) Volcanic plot showing the differences in gene expression between TCR engineered TILs vs the wild-type TILs.

(C) Heatmap of signature genes expression of different clusters.

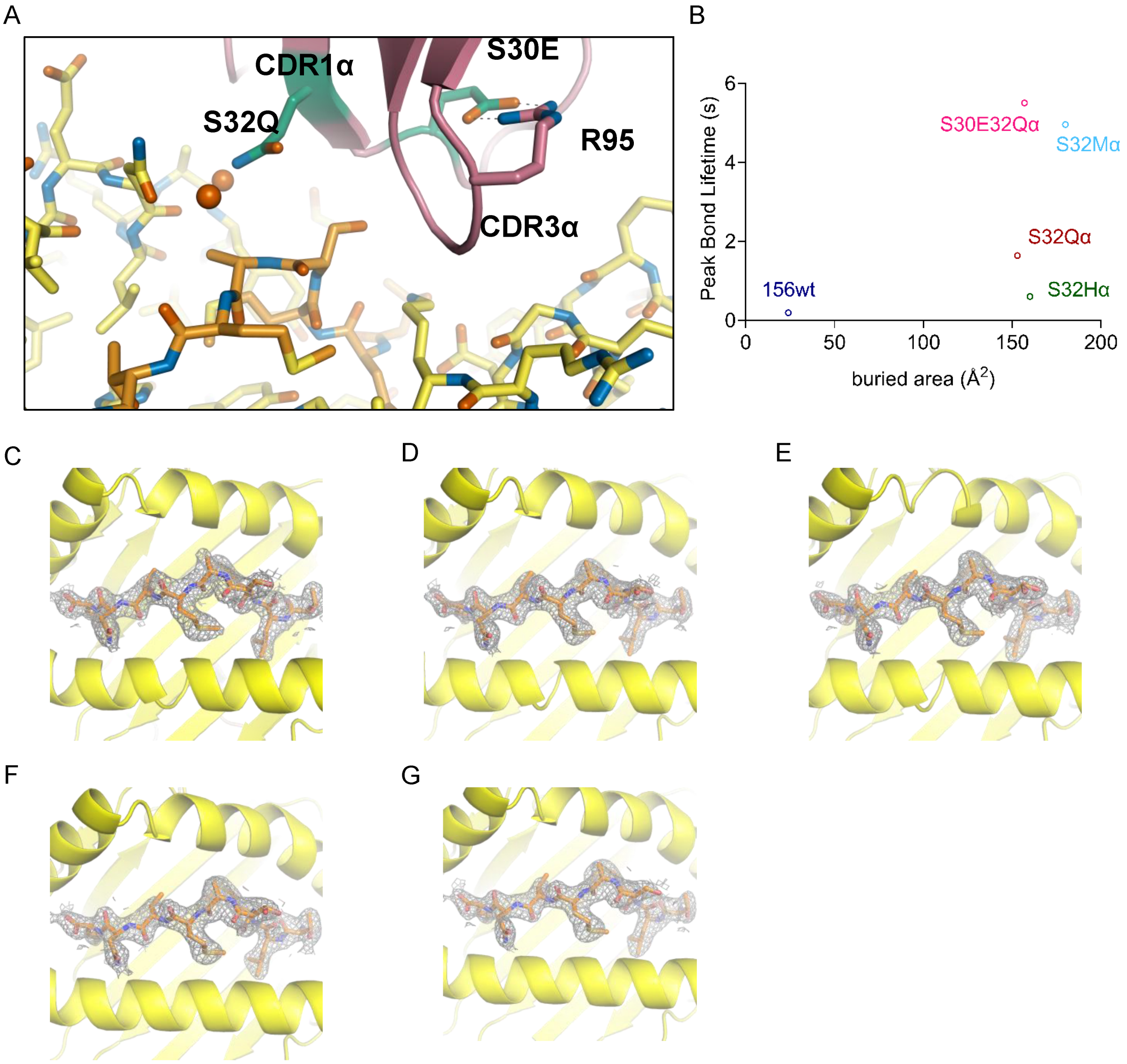

Fig. S11:

(A) Details of interaction of S30E and S32Q mutant with PAP peptide. TCR β chain is omitted for clarity. S30E is not in contact with the PAP peptide but with the CDR3 alpha chain.

(B) The bond lifetime measured by BFP did not correlate with the buried area between the Ser32 mutation and the PAP peptide.

(C-G) Simulated annealing peptide omit 2mFo-DFc maps (gray) contoured at 1-sigma around the PAP peptide (orange) in the wt (C), S32Hα (D), S32Qα (E), S32Mα (F), and S30E32Qα (G) structures. HLA:A*0201 (yellow) is shown as a cartoon; TCRs and solvent molecules are omitted for clarity.

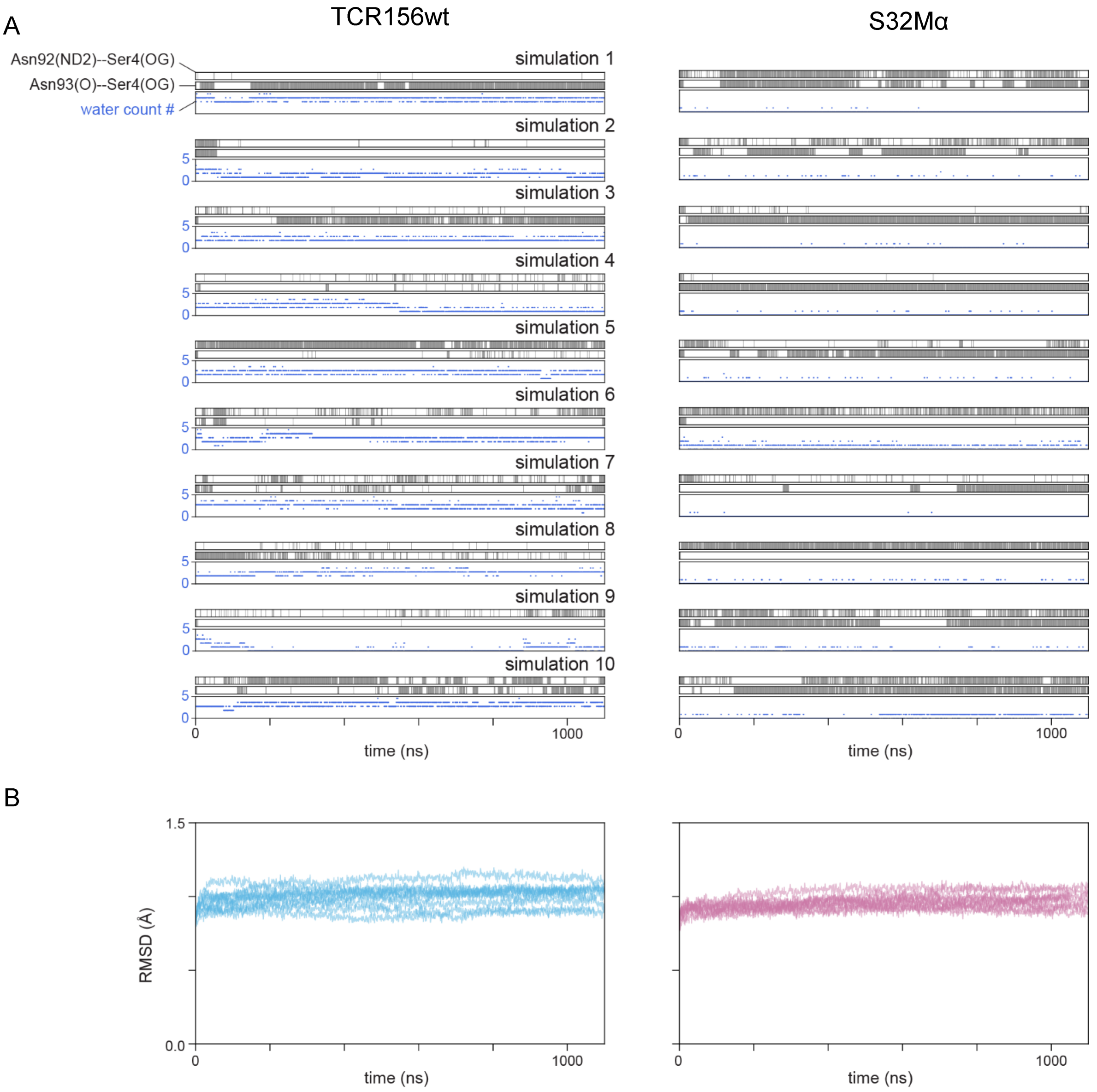

Fig. S12: MD measurements.

(A) Top and middle traces: presence or absence of S4-hydroxyl mediated hydrogen bonds formed with either N92 or N93. Bottom trace: numbers of water molecules within bounding sphere (see Methods) with y-axis values corresponding to no waters in the sphere (0) up to five waters in the sphere (5). Data represent every 1 ns of unrestrained simulation for all simulation replicates of 156wt (left) or S32Mα (right).

(B) Root-mean-square deviations (RMSD) of Cα atoms for all protein chains from each starting structure; ten traces overlaid per condition for either 156wt (left) or S32Mα (right).

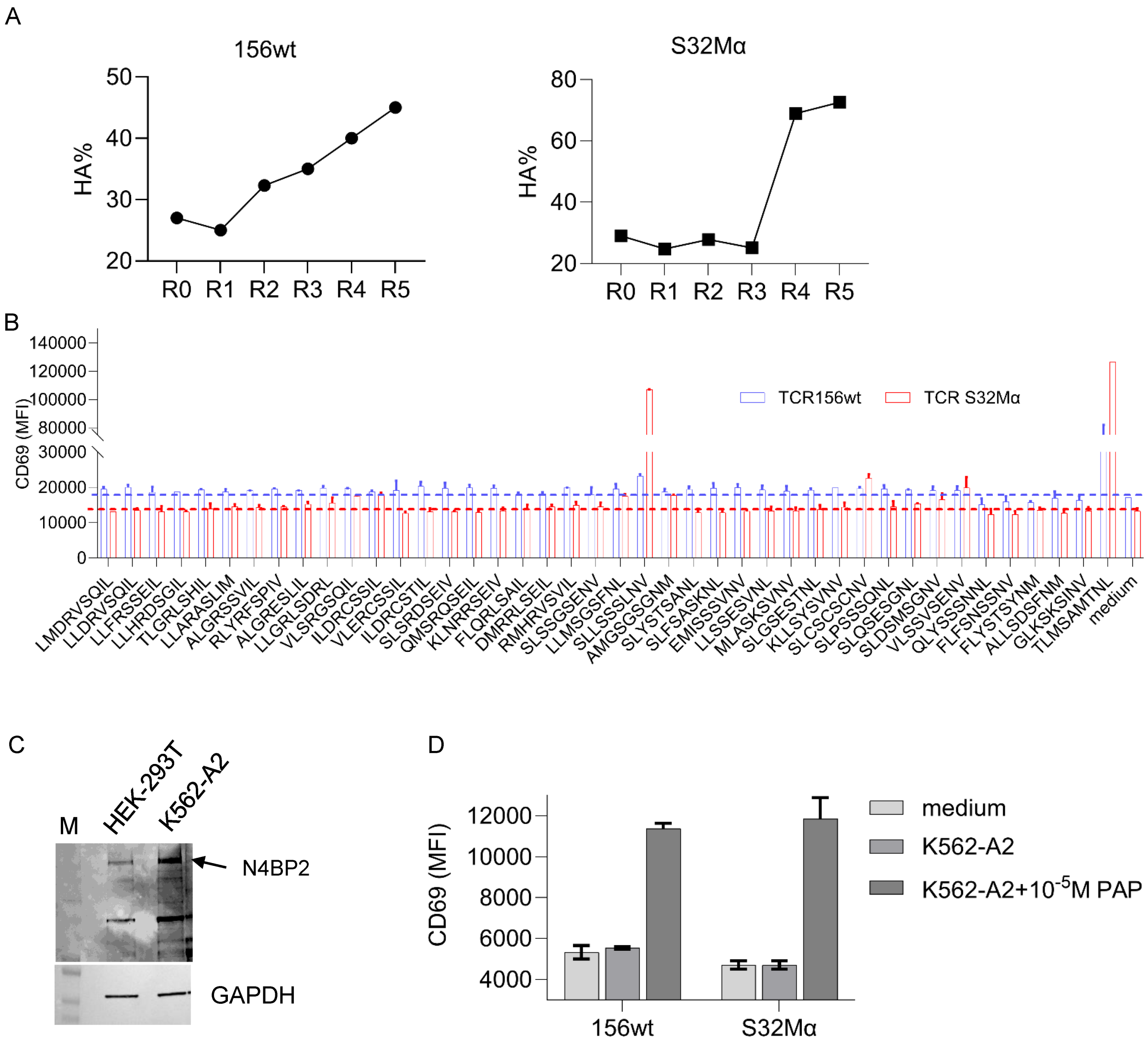

Fig. S13: Cross-reactivity analysis of the TCR156wt and S32Mα.

(A) HA marker enrichment after each round of selection by TCRwt or S32Mα tetramers.

(B) CD69 expression of the SKW-3 cells expressing TCRwt or S32Mα after co-cultured with synthesized peptides predicted from human proteome.

(C) Western blot evaluation of N4DD binding protein 2 (N4BP2) expression in different cell lines.

(D) Co-culture of SKW-3 cells expressing 156wt or S32Mα with K562-A2 with or without pulsing of PAP_{22} peptide.

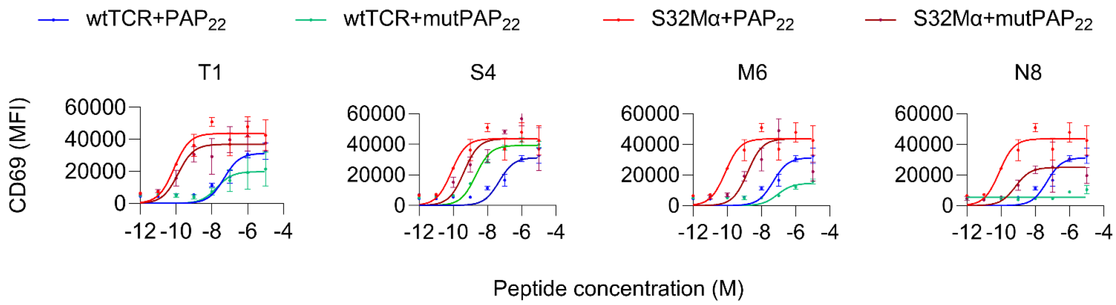

Fig. S14: Alanine scanning mutagenesis of PAP peptide recognition by TCR156wt and S32Mα.

Series of titrated alanine substituted PAP_{22} peptides were pulsed onto T2 cells and co-cultured with SKW-3 cells expressing TCRwt or S32Mα for 16 hours before CD69 was measured.

Table S1. Data collection and refinement statistics.

Structure	wt	S32Hα	S32Qα	S32Mα	S30E32Qα
Wavelength (Å)	1	1.031	1	1	1
Resolution range (Å)	46.32 - 2.1 (2.16 - 2.1)	46 - 1.97 (2.01 - 1.97)	49.66 - 2.01 (2.06 - 2.01)	46.11 - 2.19 (2.24 - 2.19)	46.02 - 2.11 (2.15 - 2.11)
Space group	C 1 2 1	C 1 2 1	C 1 2 1	C 1 2 1	C 1 2 1
Unit cell (Å, °)	225.107 47.349 90.73 90 95.83 90	228.072 47.317 92.407 90 95.36 90	225.225 46.065 91.339 90 94.8 90	225.956 46.307 91.793 90 95.13 90	226.008 46.635 92.552 90 96.08 90
Total reflections	178228 (15342)	362839 (12506)	213643 (15647)	166828 (12114)	190865 (11369)
Unique reflections	55350 (4641)	68724 (3467)	60407 (4302)	48631 (3455)	55203 (3202)
Multiplicity	3.2 (3.3)	5.3 (3.6)	3.5 (3.6)	3.4 (3.5)	3.5 (3.6)
Completeness (%)	98.39 (99.38)	97.96 (85.14)	96.06 (97.68)	98.48 (99.45)	98.73 (98.52)
Mean I/sigma(I)	7.03 (1.01)	8.97 (0.54)	9.73 (0.66)	9.32 (0.76)	10.10 (0.57)
Wilson B-factor ($Å^2$)	42.06	42.66	43.26	46.83	49.24
R-merge	0.09938 (0.9876)	0.08242 (1.689)	0.07702 (1.668)	0.09157 (1.486)	0.0752 (1.81)
R-meas	0.1197 (1.176)	0.09152 (1.971)	0.09109 (1.957)	0.109 (1.758)	0.08935 (2.135)
R-pim	0.06568 (0.6305)	0.03902 (0.9917)	0.04804 (1.015)	0.05829 (0.9287)	0.04761 (1.12)
CC1/2	0.994 (0.583)	0.998 (0.373)	0.998 (0.371)	0.997 (0.362)	0.998 (0.35)
Reflections used in refinement	55323 (4636)	68639 (3448)	60381 (4292)	48607 (3450)	55160 (3188)
Reflections used for R-free	1703 (143)	2355 (119)	2012 (156)	2000 (142)	2407 (139)
R-work	0.1937 (0.3202)	0.1796 (0.3580)	0.1958 (0.3533)	0.1932 (0.3122)	0.1915 (0.3615)
R-free	0.2251 (0.3593)	0.2203 (0.3754)	0.2290 (0.3549)	0.2288 (0.3253)	0.2338 (0.4158)
Number of non-hydrogen atoms	6973	6898	7006	6986	6909

macromolecules	6623	6622	6631	6636	6625
ligands	84	78	103	129	113
solvent	266	198	272	221	171
Protein residues	822	822	822	822	822
RMS(bonds) (Å)	0.004	0.013	0.004	0.003	0.005
RMS(angles) (°)	0.62	1.16	0.62	0.57	0.73
Ramachandran favored (%)	98.03	97.54	97.66	97.54	98.15
Ramachandran outliers (%)	0.00	0.00	0.00	0.00	0.00
Rotamer outliers (%)	2.22	3.18	2.90	0.97	2.64
Clashscore	3.37	4.06	4.19	3.94	4.34
Average B-factor (Å²)	60.39	63.84	58.90	63.38	68.61
macromolecules	60.61	63.93	59.06	63.59	68.72
ligands	70.86	77.26	69.36	70.93	80.47
solvent	51.60	55.37	50.87	52.67	56.40

Statistics for the highest-resolution shell are shown in parentheses.

References and Notes

67. A. J. Meyer, J. W. Ellefson, A. D. Ellington, Library generation by gene shuffling. Curr. Protoc. Mol. Biol., 1–7 (2014).
68. P. A. Nesterenko, J. McLaughlin, B. L. Tsai, G. Burton Sojo, D. Cheng, D. Zhao, Z. Mao, N. J. Bangayan, M. B. Obusan, Y. Su, R. H. Ng, W. Chour, J. Xie, Y. R. Li, D. Lee, M. Noguchi, C. Carmona, J. W. Phillips, J. T. Kim, L. Yang, J. R. Heath, P. C. Boutros, O. N. Witte, HLA-A∗02:01 restricted T cell receptors against the highly conserved SARS-CoV-2 polymerase cross-react with human coronaviruses. Cell Rep. 37 (2021).
69. Y. Hao, S. Hao, E. Andersen-Nissen, W. M. Mauck, S. Zheng, A. Butler, M. J. Lee, A. J. Wilk, C. Darby, M. Zager, P. Hoffman, M. Stoeckius, E. Papalexi, E. P. Mimitou, J. Jain, A. Srivastava, T. Stuart, L. M. Fleming, B. Yeung, A. J. Rogers, J. M. McElrath, C. A. Blish, R. Gottardo, P. Smibert, R. Satija, Integrated analysis of multimodal single-cell data. Cell 184, 3573-3587.e29 (2021).
70. Y. Hao, T. Stuart, M. H. Kowalski, S. Choudhary, P. Hoffman, A. Hartman, A. Srivastava, G. Molla, S. Madad, C. Fernandez-Granda, R. Satija, Dictionary learning for integrative, multimodal and scalable single-cell analysis. Nat. Biotechnol. 42, 293–304 (2024).
71. T. Stuart, A. Butler, P. Hoffman, C. Hafemeister, E. Papalexi, W. M. Mauck, Y. Hao, M. Stoeckius, P. Smibert, R. Satija, Comprehensive Integration of Single-Cell Data. Cell 177, 1888-1902.e21 (2019).
72. A. Butler, P. Hoffman, P. Smibert, E. Papalexi, R. Satija, Integrating single-cell transcriptomic data across different conditions, technologies, and species. Nat. Biotechnol. 36, 411–420 (2018).

73. R. Satija, J. A. Farrell, D. Gennert, A. F. Schier, A. Regev, Spatial reconstruction of single-cell gene expression data. Nat. Biotechnol. 33, 495–502 (2015).
74. C. Trapnell, D. Cacchiarelli, J. Grimsby, P. Pokharel, S. Li, M. Morse, N. J. Lennon, K. J. Livak, T. S. Mikkelsen, J. L. Rinn, The dynamics and regulators of cell fate decisions are revealed by pseudotemporal ordering of single cells. Nat. Biotechnol. 32, 381–386 (2014).
75. X. Qiu, Q. Mao, Y. Tang, L. Wang, R. Chawla, H. A. Pliner, C. Trapnell, Reversed graph embedding resolves complex single-cell trajectories. Nat. Methods 14, 979–982 (2017).
76. J. Cao, M. Spielmann, X. Qiu, X. Huang, D. M. Ibrahim, A. J. Hill, F. Zhang, S. Mundlos, L. Christiansen, F. J. Steemers, C. Trapnell, J. Shendure, The single-cell transcriptional landscape of mammalian organogenesis. Nature 566, 496–502 (2019).
77. L. McInnes, J. Healy, J. Melville, UMAP: Uniform Manifold Approximation and Projection for Dimension Reduction. doi: 10.48550/arXiv.1802.03426 (2018).
78. X. Yang, L. I. Garner, I. V. Zvyagin, M. A. Paley, E. A. Komech, K. M. Jude, X. Zhao, R. A. Fernandes, L. M. Hassman, G. L. Paley, C. S. Savvides, S. Brackenridge, M. N. Quastel, D. M. Chudakov, P. Bowness, W. M. Yokoyama, A. J. McMichael, G. M. Gillespie, K. C. Garcia, Autoimmunity-associated T cell receptors recognize HLA-B*27-bound peptides. Nature 612, 771–777 (2022).
79. W. Kabsch, XDS. Acta Crystallogr. Sect. D Biol. Crystallogr. 66, 125–132 (2010).
80. M. D. Winn, C. C. Ballard, K. D. Cowtan, E. J. Dodson, P. Emsley, P. R. Evans, R. M. Keegan, E. B. Krissinel, A. G. Leslie, A. McCoy, S. J. McNicholas, G. N. Murshudov, N. S. Pannu, E. A. Potterton, H. R. Powell, R. J. Read, A. Vagin, K. S. Wilson, Overview of the CCP4 suite and current developments. Acta Crystallogr. Sect. D Biol. Crystallogr. 67, 235–242 (2011).
81. P. R. Evans, G. N. Murshudov, How good are my data and what is the resolution? Acta Crystallogr. Sect. D 69, 1204–1214 (2013).
82. A. J. McCoy, R. W. Grosse-Kunstleve, P. D. Adams, M. D. Winn, L. C. Storoni, R. J. Read, Phaser crystallographic software. J. Appl. Cryst 40, 658–674 (2007).
83. T. C. Terwilliger, R. W. Grosse-Kunstleve, P. V Afonine, N. W. Moriarty, P. H. Zwart, L.-W. Hung, R. J. Read, P. D. Adams, Iterative model building, structure refinement and density modification with the PHENIX AutoBuild wizard. Acta Crystallogr D Biol Crystallogr 64, 61–69 (2008).
84. P. Emsley, B. Lohkamp, W. G. Scott, K. Cowtan, Features and Development of Coot. Acta Crystallogr D Biol Crystallogr 66, 486–501 (2010).
85. N. Echols, R. W. Grosse-Kunstleve, P. V Afonine, G. Bunkóczi, V. B. Chen, J. J. Headd, A. J. McCoy, N. W. Moriarty, R. J. Read, D. C. Richardson, J. S. Richardson, T. C. Terwilliger, P. D. Adams, Graphical tools for macromolecular crystallography in PHENIX. J. Appl. Cryst 45, 581–586 (2012).
86. P. V Afonine, R. W. Grosse-Kunstleve, N. Echols, J. J. Headd, N. W. Moriarty, M. Mustyakimov, T. C. Terwilliger, A. Urzhumtsev, P. H. Zwart, P. D. Adams, Towards automated crystallographic structure refinement with phenix.refine. Acta Crystallogr D Biol Crystallogr 68, 352–367 (2012).
87. D. Liebschner, P. V Afonine, M. L. Baker, G. Bunkóczi, V. B. Chen, T. I. Croll, B. Hintze, L. W. Hung, S. Jain, A. J. McCoy, N. W. Moriarty, R. D. Oeffner, B. K. Poon, M. G. Prisant, R. J. Read, J. S. Richardson, D. C. Richardson, M. D. Sammito, O. V Sobolev, D. H. Stockwell, T. C. Terwilliger, A. G. Urzhumtsev, L. L. Videau, C. J. Williams, P. D. Adams, Macromolecular structure determination using X-rays, neutrons and electrons: recent developments in Phenix. Acta Crystallogr. Sect. D, Struct. Biol. 75, 861–877 (2019).

88. V. B. Chen, W. B. Arendall, J. J. Headd, D. A. Keedy, R. M. Immormino, G. J. Kapral, L. W. Murray, J. S. Richardson, D. C. Richardson, MolProbity: all-atom structure validation for macromolecular crystallography. Acta Crystallogr. Sect. D Biol. Crystallogr. 66, 12–21 (2010).
89. A. Morin, B. Eisenbraun, J. Key, P. C. Sanschagrin, M. A. Timony, M. Ottaviano, P. Sliz, Cutting edge: Collaboration gets the most out of software. Elife 2 (2013).
90. M. H. Gee, A. Han, S. M. Lofgren, S. R. Quake, M. M. Davis, K. C. Garcia, M. H. Gee, A. Han, S. M. Lofgren, J. F. Beausang, J. L. Mendoza, M. E. Birnbaum, R. A. Fernandes, A. Velasco, D. Baltimore, T. N. Schumacher, P. Khatri, S. R. Quake, Antigen identification for orphan T cell receptors expressed on tumor-infiltrating lymphocytes. Cell 172, 549–556 (2018).
91. D. A. Case, H. M. Aktulga, K. Belfon, D. S. Cerutti, G. A. Cisneros, V. W. D. Cruzeiro, N. Forouzesh, T. J. Giese, A. W. Götz, H. Gohlke, S. Izadi, K. Kasavajhala, M. C. Kaymak, E. King, T. Kurtzman, T.-S. Lee, P. Li, J. Liu, T. Luchko, R. Luo, M. Manathunga, M. R. Machado, H. M. Nguyen, K. A. O'Hearn, A. V. Onufriev, F. Pan, S. Pantano, R. Qi, A. Rahnamoun, A. Risheh, S. Schott-Verdugo, A. Shajan, J. Swails, J. Wang, H. Wei, X. Wu, Y. Wu, S. Zhang, S. Zhao, Q. Zhu, T. E. I. Cheatham, D. R. Roe, A. Roitberg, C. Simmerling, D. M. York, M. C. Nagan, K. M. Jr. Merz, AmberTools. J. Chem. Inf. Model. 63, 6183–6191 (2023).
92. C. Tian, K. Kasavajhala, K. A. A. Belfon, L. Raguette, H. Huang, A. N. Migues, J. Bickel, Y. Wang, J. Pincay, Q. Wu, C. Simmerling, ff19SB: Amino-Acid-Specific Protein Backbone Parameters Trained against Quantum Mechanics Energy Surfaces in Solution. J. Chem. Theory Comput. 16, 528–552 (2020).
93. S. Izadi, R. Anandakrishnan, A. V. Onufriev, Building Water Models: A Different Approach. J. Phys. Chem. Lett. 5, 3863–3871 (2014).
94. D. A. Pearlman, D. A. Case, J. W. Caldwell, W. S. Ross, T. E. Cheatham, S. DeBolt, D. Ferguson, G. Seibel, P. Kollman, AMBER, a package of computer programs for applying molecular mechanics, normal mode analysis, molecular dynamics and free energy calculations to simulate the structural and energetic properties of molecules. Computer Physics Communications 91, 1–41 (1995).
95. R. Salomon-Ferrer, A. W. Götz, D. Poole, S. Le Grand, R. C. Walker, Routine Microsecond Molecular Dynamics Simulations with AMBER on GPUs. 2. Explicit Solvent Particle Mesh Ewald. J. Chem. Theory Comput. 9, 3878–3888 (2013).
96. J.-P. Ryckaert, G. Ciccotti, H. J. C. Berendsen, Numerical integration of the cartesian equations of motion of a system with constraints: molecular dynamics of n-alkanes. Journal of Computational Physics 23, 327–341 (1977).
97. C. W. Hopkins, S. Le Grand, R. C. Walker, A. E. Roitberg, Long-Time-Step Molecular Dynamics through Hydrogen Mass Repartitioning. J. Chem. Theory Comput. 11, 1864–1874 (2015).
98. W. Humphrey, A. Dalke, K. Schulten, VMD: Visual molecular dynamics. Journal of Molecular Graphics 14, 33–38 (1996).
99. N. Michaud-Agrawal, E. J. Denning, T. B. Woolf, O. Beckstein, MDAnalysis: A toolkit for the analysis of molecular dynamics simulations. Journal of Computational Chemistry 32, 2319–2327 (2011).
